# A Naphthoquinoline-Dione-Based Cu^2+^ Sensing Probe with Visible Color Change and Fluorescence Quenching in an Aqueous Organic Solution

**DOI:** 10.3390/molecules29040808

**Published:** 2024-02-09

**Authors:** Ashwani Kumar, Pil Seok Chae

**Affiliations:** Department of Bionano Engineering, Hanyang University, Ansan 15588, Republic of Korea

**Keywords:** fluorescence, naphthoquinoline-dione, Cu^2+^ sensing, colorimetric/ratiometric

## Abstract

Copper metal ions (Cu^2+^) are widely used in various industries, and their salts are used as supplementary components in agriculture and medicine. As this metal ion is associated with various health issues, it is necessary to detect and monitor it in environmental and biological samples. In the present report, we synthesized a naphthoquinoline-dione-based probe **1** containing three ester groups to investigate its ability to detect metal ions in an aqueous solution. Among various metal ions, probe **1** showed a vivid color change from yellow to colorless in the presence of Cu^2+^, as observed by the naked eye. The ratiometric method using the absorbance ratio (***A***_413_/***A***_476_) resulted in a limit of detection (LOD) of 1 µM for Cu^2+^. In addition, the intense yellow-green fluorescence was quenched upon the addition of Cu^2+^, resulting in a calculated LOD of 5 nM. Thus, probe **1** has the potential for dual response toward Cu^2+^ detection through color change and fluorescence quenching. ^1^H-NMR investigation and density functional theory (DFT) calculations indicate 1:1 binding of the metal ion to the small cavity of the probe comprising four functional groups: the carbonyl group of the amide (O), the amino group (N), and two *t*-butyl ester groups (O). When adsorbed onto various solid surfaces, such as cotton, silica, and filter paper, the probe showed effective detection of Cu^2+^ via fluorescence quenching. Probe **1** was also useful for Cu^2+^ sensing in environmental samples (sea and drain water) and biological samples (live HeLa cells).

## 1. Introduction

Potential adaptability, easy synthesis and structural modification, and outstanding photo-physical properties of small molecular probes led to their wide applications in various fields, including medicinal chemistry, bio-imaging, drug delivery, solar cells, and OLEDs [1,2,3,4]. Recently, significant attention has been paid to molecular probes that exhibit a vibrant color change in the presence of specific analytes [5,6,7,8]. Metal ion sensing in aqueous systems is particularly demanding due to the critical roles of various metal ions in physiology. However, at their individual optimal concentrations, these metal ions perform diverse functions, including pH regulation, osmotic balance, metabolism, and signaling transduction [9,10]. Their abnormal concentrations could lead to damage of organs that can eventually show detrimental effects on the human body, such as physical disorders and acute/chronic diseases [11,12,13]. Heavy metal ions, such as Hg^2+^/CH_3_Hg^+^ and Pb^2+^, are toxic to humans [14,15]. Thus, selective detection of individual metal ions is indispensable, and monitoring the quantity/concentration of these metal ions in environmental sources is required and should be regulated, especially in drinking water (e.g., tap/lake water) [16,17,18,19]. Cu^2+^ is the third most common heavy metal ion in the human body and functions to sustain bones, blood vessels, the immune system, nerves, and metabolism, and it serves a critical function in iron absorption in the human body [20]. However, excessive Cu^2+^ exposure, even for a limited time, can lead to serious health concerns, such as liver/kidney issues, gastrointestinal (GI) dysfunction, and many serious neurodegenerative diseases (e.g., Menkes disease) [21,22,23,24,25]. It is also widely used in construction, transport, and electronics, and many of its salts are being used as supplementary components in agriculture and medicine [26,27]. This metal ion seeps into groundwater and deteriorates the quality of drinking water, posing a threat to human health. The U.S. Environmental Protection Agency (EPA) [28] currently sets the limit of Cu^2+^ concentration in drinking water at 1.3 ppm (~20 µM).

Among various analytical techniques to detect metal ions in biological and environmental samples [29], fluorescence spectroscopy possesses many advantages over others in terms of affordability, quick response, convenience, easy operation, on-site detection/monitoring, and naked eye recognition [30,31,32,33,34,35]. Thus, many researchers have made efforts to create selective, reliable, convenient, and efficient sensors with a variety of fluorescent platforms, such as fluorescein, rhodamine B, triphenylamine, quinolone, pyrene, anthraquinone, chalcone, benzothiazole/benzimidazole amine, coumarin-conjugated di-(2-picolyl)amine, peptide, and Schiff base compounds [36,37,38,39,40,41,42,43]. Many of these probes utilized ethylene diamine-like structures for strong and selective Cu^2+^ binding among different metal ions, leading to high selectivity toward Cu^2+^ detection. For instance, S. Anbu et al. reported a benzimidazole-based bis-Schiff base probe as a fluorogenic chemosensor. The Schiff base-benzimidazole conjugate mimics ethylene diamine as a metal ion-binding group. This probe detected Cu^2+^ and Zn^2+^ in an aqueous solution via fluorescence ‘turn-on’ and ‘turn-off’ mechanisms, respectively, with limits of detection (LODs) of 24 and 2 nM [36]. K. Guo and J. Li used an imine-linked triphenylamine–benzothiazole conjugate as a metal ion-sensing probe. The probe containing an ethylenediamine-like binding site for metal ions was able to detect two metal ions (Cu^2+^ and Fe^3+^) in CH_3_CN, with LODs of 0.3 and 3 μM, respectively [37]. The probe showed a color change from colorless to red in the presence of Cu^2+^, while the interaction of the probe with Fe^3+^ resulted in the ‘switch on’ fluorescence emission. In an aqueous CH_3_CN solution, this benzothiazole-based probe was selective for Zn^2+^, with an LOD of 3 μM. D. Aydin et al. reported a phenolphthalein-based Schiff base probe for selective Cu^2+^ detection. The metal binding site of the probe is structurally similar to ethylenediamine but comprises a different spacer (propylene) and atom (oxygen) from the latter. Upon the addition of the metal ion, the probe displayed a color change from yellow to colorless in an HEPES:CH_3_CN (1:1, pH = 7) solution. In addition, the fluorescence emission of the probe turned off in the presence of Cu^2+^, giving an LOD of 84 nM [38]. G. Singh et al. reported a pyrene-bearing organosilane-based nanoparticle for the selective detection of Cu^2+^. This pyrene-decorated nanoparticle also contains an ethylenediamine-like binding site and showed a sensitive Cu^2+^ detection (LOD = 6 µM) in methanol [43]. Inspired by the structural feature of such ethylenediamine-like binding sites present in these previously reported probes, we prepared a new probe (probe **1**) containing three ester groups based on the naphthoquinoline-dione platform in the current study. The resulting three ester group probe selectively detected Cu^2+^ by luminescence/fluorescence and absorption/color change. The probe **1** solution changed from bright yellow to colorless upon Cu^2+^ binding, demonstrating a low calculated LOD of 5 nM. Cu^2+^ sensing was also realized using this probe adsorbed onto solid surfaces (cotton, filter paper, silica gel, and TLC plate). In addition, Cu^2+^ added in environmental samples (sea/drain water) or bio-samples (HeLa cells) was successfully detected by the probe.

## 2. Results and Discussion

### 2.1. Synthesis and Characterization of Probe ***1***

**Scheme 1:**
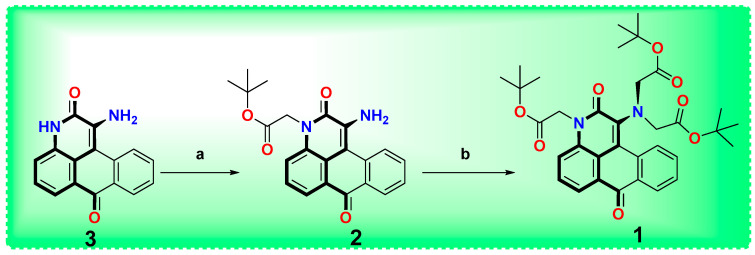
Synthetic scheme of probe **1**: (**a**) K_2_CO_3_, DMSO, BrCH_2_COO^t^Bu, TBAHSO_4_, RT, 2 days, and (**b**) BrCH_2_COO^t^Bu, K_2_CO_3_, THF, TBAHSO_4_, RT, 3 days.

**Figure 1:**
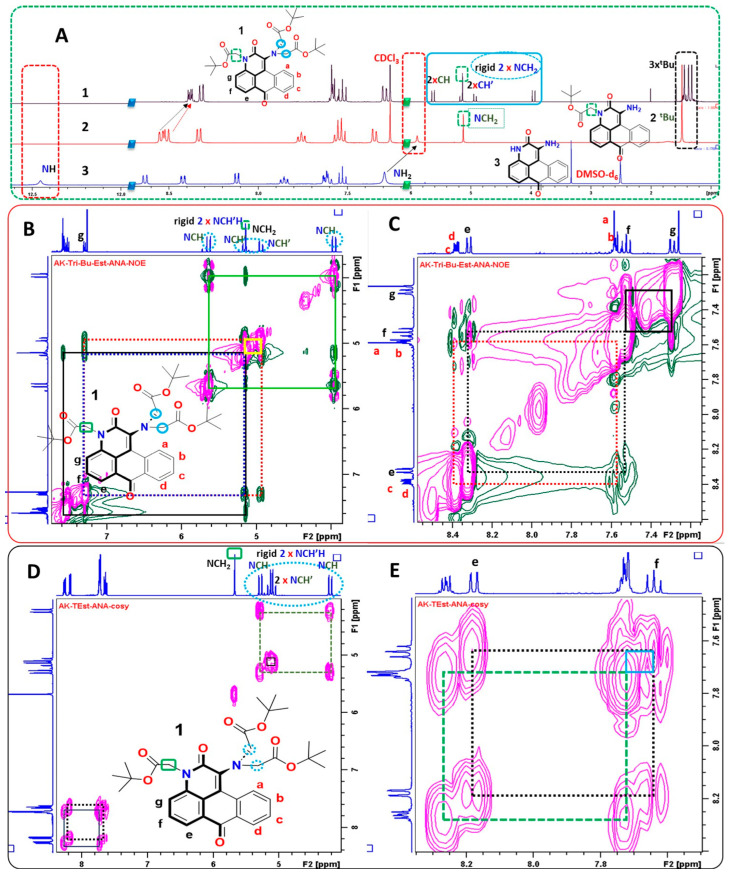
(**A**) Partial ^1^H NMR spectra of probe **1** and compounds **2** and **3** with assignments of the main peaks. CDCl_3_ was used as the NMR solvent for probe **1** and compound **2**, while DMSO-*d*_6_ was used for probe **3**. (**B**,**C**) The partial ^1^H-^1^H NOESY spectrum of probe **1** dissolved in CDCl_3_ and (**D**,**E**) the ^1^H-^1^H COSY spectrum of probe **1** dissolved in DMSO-*d*_6_. The letters a–g in (**C**,**E**) correspond to the aromatic protons of probe **1**, as indicated in the chemical structure in (**B**). Strong correlations between the two neighboring protons are indicated by the colored square boxes.

Probe **1** was synthesized from compound **3** via a two-step reaction (Scheme 1). The amino group bearing the naphthoquinoline-dione derivative (compound **3**) was reacted with *t*-butyl bromoacetate in DMSO using K_2_CO_3_ (base) and TBAHSO_4_ (catalyst) to produce compound **2** (50%). The formation of compound **2** was verified by its ^1^H-NMR spectrum, where the amidic NH of compound **3** was lost at 12.45 ppm, and two new singlets of NCH_2_ and *t*-butyl groups appeared in the aliphatic region at δ 5.13 and 1.49 ppm, respectively (Figure 1A). A further reaction of compound **2** with *t*-butyl bromoacetate gave probe **1** a moderate yield (50%). The ^1^H NMR spectrum of the probe showed a loss of the NH_2_ signal at δ 5.89 ppm with the simultaneous appearance of four doublets at δ 3.95, 4.93, 5.16, and 5.63 ppm, corresponding to two CH_2_ groups attached to the amino group. These spectral features match the chemical structure of probe **1** containing three *t*-butyl acetate groups (Figure 1A). The 2D NMR (^1^H-^1^H NOESY and COSY) spectra verify the chemical structure of probe **1** (Figure 1B–E and Figures S1–S4). The 2D NMR (^1^H-^1^H NOESY) spectrum displayed a strong correlation between the three *t*-butyl protons, their neighboring protons (N*^α^*CH_2_), and the nearest aromatic protons (Figures S1 and S2). The CH_2_ attached to the amidic N correlates with the aromatic proton (H_g_), while two rigid CH_2_ groups attached to the amino N strongly correlate with the nearby aromatic proton (H_a_) (Figure 1B,C). Further correlations were observed between H_g_ (doublet) and H_f_ (triplet), as well as between H_f_ (triplet) and H_e_ (doublet) (Figure 1B,C). The 2D NMR (^1^H-^1^H COSY) spectrum of the probe revealed correlations between two neighboring protons (Figure 1D,E and Figures S3–S5). The ^13^C NMR and HRMS spectra further confirmed the identity of the probe (see electronic Supplementary Materials).

### 2.2. Spectroscopic Characterization and Sensing Ability of Probe ***1***

**Figure 2:**
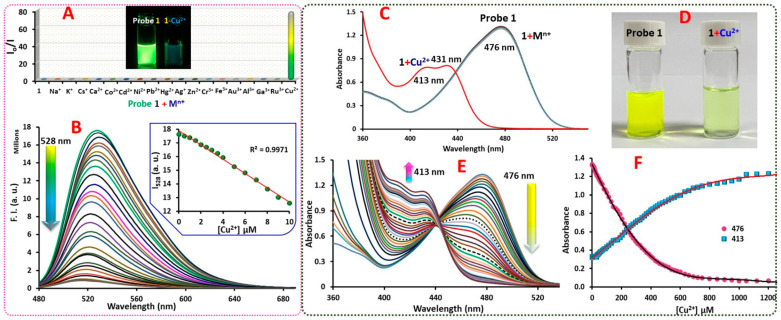
(**A**) Bar diagram showing the changes in fluorescence intensity of probe **1** (5 µM, ACN) upon the addition of different metal ions. The fluorescence intensity changes are represented as fluorescence intensity ratios (***I***_o_/***I***), where ***I*** and ***I***_o_ are the fluorescence intensities of the probe at 528 nm in the presence and absence of individual metal ions, respectively. The inset shows luminescence quenching of the probe **1** solution upon the addition of Cu^2+^. A Cu^2+^ stock solution was prepared by dissolving Cu(ClO_4_)_2_·6H_2_O in ACN. (**B**) Fluorescence spectral changes during the titration of probe **1** with Cu^2+^. The inset shows a linear correlation between fluorescence intensity at 528 nm (***I***_528_) and [Cu^2+^] in a low [Cu^2+^] range (0–10 µM). The points and line represent the experimental values and curved fit, respectively, where *λ_ex_* = 470 nm. (**C**) UV–Vis absorption spectral changes of probe **1** (50 µM, ACN) upon the addition of various metal ions individually. (**D**) Image of the probe solution showing a color change from yellow-green to pale green upon the addition of Cu^2+^ in daylight, as detected by the naked eye. (**E**) UV–Vis absorption spectral changes during the titration of probe **1** (50 µM, ACN) with Cu^2+^ and (**F**) changes in absorption values at both 476 and 413 nm (***A***_476_ and ***A***_413_, respectively) with increasing [Cu^2+^]. The points and line represent experimental values and non-linear curve fitting, respectively.

To explore the probe potential in metal ion sensing, we performed fluorescence studies of probe **1** in acetonitrile (ACN)). In the range of excitation wavelengths from 390 to 500 nm, the probe exhibited maximum emission or the highest quantum yield (ø = 0.45) at 470 nm (Figure S6). Thus, this excitation wavelength was chosen for further fluorescence studies of the probe. Different trivalent (Cr^3+^, Fe^3+^, Ga^3+^, Al^3+^, and Ru^3+^), divalent (Mg^2+^, Ca^2+^, Ba^2+^, Fe^2+^, Co^2+^, Zn^2+^, Cd^2+^, Pb^2+^, Cu^2+^, Ni^2+^, and Hg^2+^), and monovalent metal ions (Ag^+^, Cs^+^, Na^+^, and K^+^) were added individually to the ACN solution containing probe **1** at 5 µM. Among these metal ions, only Cu^2+^ was able to selectively quench the green luminescence of probe **1** (Figure S7A,B and Figure 2A). Of note, compound **3**, a starting material for the preparation of probe **1**, was previously found to be Hg^2+^ selective [44]. Interestingly enough, the structural modification from compound **3** to probe **1** changed the probe selectivity toward Cu^2+^ from Hg^2+^. In the titration, upon the sequential addition of Cu^2+^ in ACN, the fluorescence intensity of probe **1** (5 µM, ACN) was reduced regularly and became saturated at about 60 µM [Cu^2+^] (Figure 2B and Figure S7C). This titration data indicate 1:1 stoichiometry between probe **1** and the metal ion with a high association constant (*K_a_* = 3.98 × 10^4^ M^−1^) (Figure S7C). The fluorescence emission intensity at 528 nm (***I*_528_**) linearly decreased with increasing [Cu^2+^], demonstrating an LOD of 0.5 µM for this sensing system (inset of Figure 2B). In addition, no significant interference was observed from other metal ions (Figure S7D). 

The UV–Vis spectrum of probe **1** (50 µM, ACN) exhibited a strong band at 476 nm, likely originating from the n → π* transition of the naphthoquinoline-dione system. Among the various metal ions studied, the addition of only Cu^2+^ resulted in a significant change in the UV–Vis absorption spectrum. The absorbance maximum was blue shifted from 476 to 413 nm, along with the appearance of a shoulder at 431 nm (Figure 2C). Upon the addition of Cu^2+^, the probe **1** solution changed from yellow-green to pale green in daylight, as detected by the naked eye and reflected in the absorption spectra obtained under the same conditions (Figure 2C,D). Over the course of UV–Vis titration carried out in ACN, the absorption band of probe **1** at 476 nm decreased with an increasing amount of Cu^2+^. At the same time, a new band of the probe at 413 nm appeared, with an isosbestic point observed at 445 nm Figure 2E,F). The absorption spectrum of the probe failed to result in further changes when more than 10 equiv. of Cu^2+^ was added to the probe solution. Non-linear regression analysis of this titration data suggests the formation of a 1:1 complex between the probe and the metal ion (*K_a_* = 3.41 × 10^3^ M^−1^) (Figure 2F). The linear relationship between ***A***_476_ and [Cu^2+^] observed at the initial stage of the titration was used to obtain an LOD of 1 µM for the sensing system (Figure S8A). A ratiometric approach using the ratio of two absorption values (***A***_413_/***A***_476_) allowed us to detect Cu^2+^ over a wide range of concentrations (1 µM to 1 mM), with an observed LOD of 1 µM (Figure S8B). The probe **1** solutions containing different metal ions showed no noticeable alteration in absorption spectra upon the addition of Cu^2+^, indicating that these metal ions did not interfere with the Cu^2+^ detection of the probe (Figure S9). In addition, the reversibility of probe **1** in Cu^2+^ binding was demonstrated by the use of EDTA (disodium ethylene-diamine tetra-acetic acid) as a metal ion chelator. The green luminescence observed for the free probe was fully recovered after adding EDTA to the [probe **1**-Cu^2+^] solution (Figure S10).

**Figure 3:**
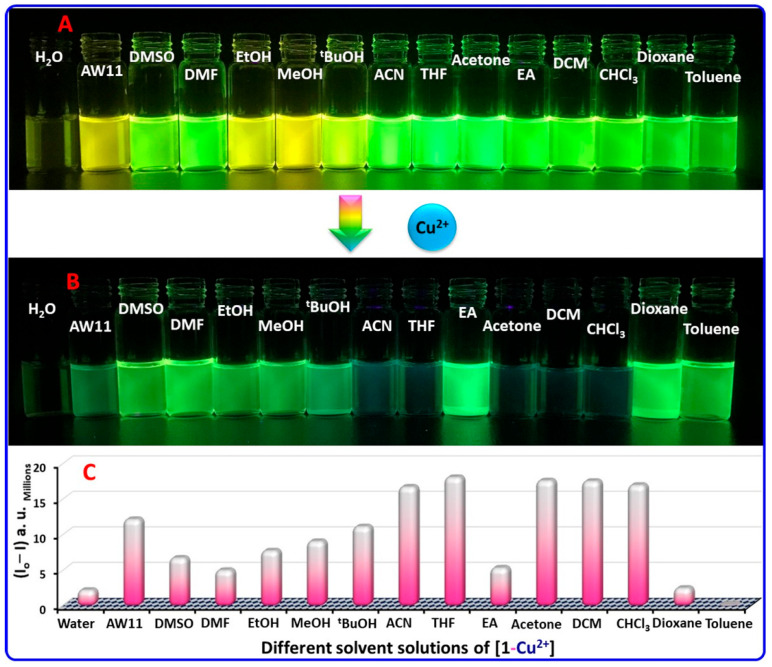
(**A**,**B**) Luminescence image of probe **1** solutions (5 µM) prepared using the various solvents under 365 nm irradiation before and after the addition of Cu^2+^ (20 equiv.) and (**C**) changes in fluorescence emission intensity (***I***_o_ − ***I***) of probe **1** (5 µM) dissolved in various solvents upon the addition of Cu^2+^ (20 equiv.), where *λ_ex_* = 470 nm. ***I***_o_ and ***I*** are the fluorescence intensities of probe **1** at the maximum emission wavelength before and after the addition of Cu^2+^, respectively. A Cu^2+^ stock solution was prepared by dissolving Cu(ClO_4_)_2_·6H_2_O in ACN.

**Figure 4:**
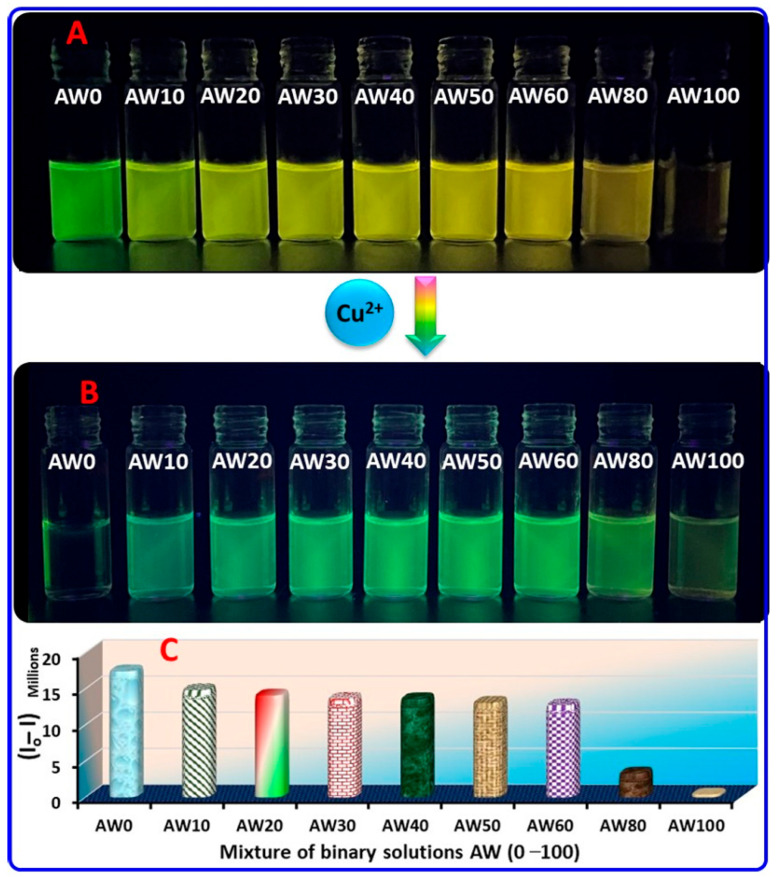
(**A**,**B**) Luminescence images obtained under illumination at 365 nm and of the probe **1** solution (5 µM) in a binary mixture of ACN and water. Luminescence images of the probe **1** solution in the absence (**A**) and presence of Cu^2+^ (20 equiv.) A Cu^2+^ stock solution was prepared by dissolving Cu(ClO_4_)_2_·6H_2_O in water. A, W, and the numbers in the vial designations indicate ACN, water, and water percentages in the solutions, respectively, where *λ_ex_* = 470 nm. (**C**) Changes in fluorescence emission intensity (***I***_o_ − ***I***) of the probe **1** solution (5 µM) at the maximum emission wavelength (*λ_em_*) upon the addition of 20 equiv. of Cu^2+^. ***I***_o_ and ***I*** represent the fluorescence intensities of the probe at *λ_em_* before and after the addition of Cu^2+^ ions, respectively.

To evaluate the solvent effects on the probe’s ability to sense Cu^2+^, we prepared probe **1** solutions at 5 µM using various solvents and added 20 equiv. of Cu^2+^ to each (Figure 3 and Figure S11). The yellow-greenish luminescence was significantly quenched (60 to 98%) for the probe dissolved in ACN, THF, acetone, DCM, or CHCl_3_. Upon the addition of Cu^2+^, the probe dissolved in EtOH, MeOH, ^t^BuOH, or AW11 (ACN/water (1:1)) resulted in moderate luminescence quenching, whereas little change in luminescence intensity was observed for the probe in pure water, dioxane, or toluene (Figure 3C). To explore the potential utility in real sample applications, we investigated the Cu^2+^-induced changes in photoluminescence and fluorescence of the probe using a binary solvent system of ACN (A) and water (W) (Figure 4 and Figure S12). With water percentage increasing up to 60% in ACN, the fluorescence emission peak of probe **1** gradually red shifted from 528 nm (green) to 567 nm (yellow), with slight decreases in emission intensity (Figure 4A and Figure S12A). When 80% water in ACN was used as a medium, we found a 60% decrease in the fluorescence intensity of probe **1** compared to that in pure ACN. This could be due to the occurrence of substantial probe aggregation, as expected from the presence of three bulky *t*-butyl groups in the probe. Upon the addition of Cu^2+^, significant fluorescence quenching was observed for probe **1** dissolving in the different binary solvents (Figure 4B and Figure S12B). This quenching effect on probe fluorescence became less obvious when the water content was greater than 80% in ACN (Figure 4C). Thus, Cu^2+^ can be effectively detected via fluorescence quenching when the water content is less than 80% in ACN. We chose 20% aqueous ACN as a binary solvent system for further studies of the probe. 

**Figure 5:**
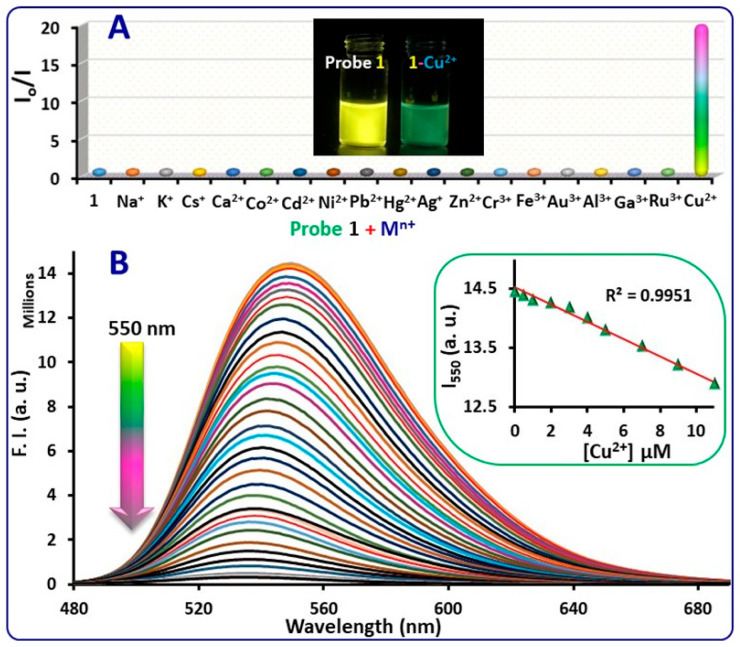
(**A**) Bar diagram showing changes in fluorescence intensity of probe **1** (5 µM, HEPES (pH 7.4): ACN = 1:4)) upon individual additions of various metal ions. The inset shows the solution color in the absence and presence of Cu^2+^. A Cu^2+^ stock solution was prepared by dissolving Cu(ClO_4_)_2_·6H_2_O in water. (**B**) Fluorescence titration of probe **1** with Cu^2+^ when excited at 470 nm. The inset shows a linear correlation between ***I***_550_ and [Cu^2+^] at a low metal ion concentration range (0–10 µM).

Upon excitation at 470 nm, the probe **1** solution (5 µM, HEPES (pH 7.4): ACN = (1:4)) exhibited an intense yellow fluorescence emission at 550 nm, along with a good quantum yield (*Φ* = 0.40) (Figure S13). Among the various metal ions, the probe responded only to Cu^2+^; the yellow luminescence and fluorescence emission of probe **1** were quenched (Figure 5A). Titration with Cu^2+^ showed a gradual decrease in fluorescence emission intensity of the probe at 550 nm (***I***_550_) with increasing [Cu^2+^] (Figure 5B and Figure S14). The titration data (***I***_550_ vs. [Cu^2+^]) are consistent with a 1:1 complex formation between probe **1** and Cu^2+^ with an association constant (*K_a_* = 1.91 × 10^4^ M^−1^). This association constant corresponds to nearly half of that observed for the [probe **1**-Cu^2+^] complex in ACN (Figure S14), indicating weaker binding of the metal ion to the probe in aqueous ACN solutions compared to pure ACN. The probe detected Cu^2+^ at concentrations as low as 0.5 µM, while the calculated LOD was 5 nM (inset of Figure 5B and Figure S15). This LOD is comparable to or better than those of many recently reported Cu^2+^-sensing probes (Table S1). We further applied the probe for metal ion sensing in seawater and drain water from a small lake, which showed the detection of Cu^2+^ as low as 0.5 µM. A slight decrease was found in probe sensitivity for the application (Figure S16). 

### 2.3. Mechanism of Cu^2+^ Interaction with Probe ***1***

**Figure 6:**
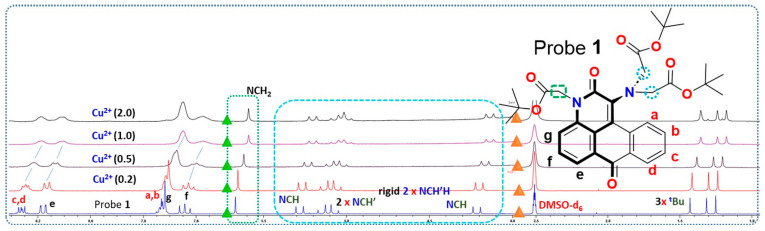
Changes in ^1^H NMR spectra (partial) of probe **1** during titration with Cu^2+^(ClO_4_)_2_·6H_2_O in DMSO-*d*_6_. The letters (a–g) represent the aromatic protons of probe **1**, as indicated in the chemical structure).

To explore the molecular interaction between probe **1** and Cu^2+^, we conducted ^1^H NMR titration of the probe with Cu^2+^ in DMSO-*d*_6_. With a gradual increase in Cu^2+^ concentration from 0 to 1 equiv., most of the probe signals (e.g., N(CH_2_) signals) were up-field shifted, along with notable broadening of the aromatic proton peaks (H_a_, H_f_, and H_e_). These changes are likely due to the paramagnetic character of Cu^2+^ combined with probe aggregation upon binding to the metal ion (Figure 6 and Figure S17). This result indicates that the tertiary N of naphthoquinoline-dione and the carbonyl (-CO) oxygen of the ester group coordinate in Cu^2+^ binding. Further addition of Cu^2+^ up to 2 equiv. failed to produce a further change in the ^1^H NMR signals, ensuring the 1:1 complex formation between the probe and Cu^2+^. 

**Scheme 2:**
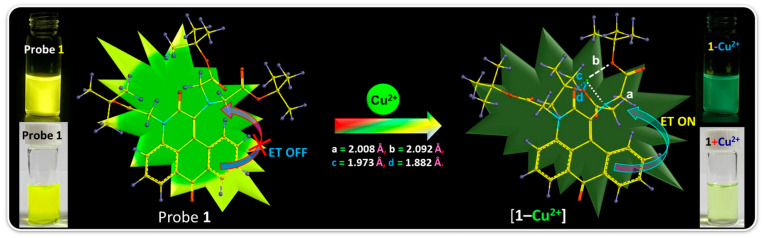
Schematic representation of a plausible mechanism responsible for the fluorescence quenching of probe **1** upon interaction with Cu^2+^. The Cu^2+^ binding to the probe allows electron transfer from the naphthoquinoline-dione ring to the metal binding site, leading to fluorescence quenching.

**Figure 7:**
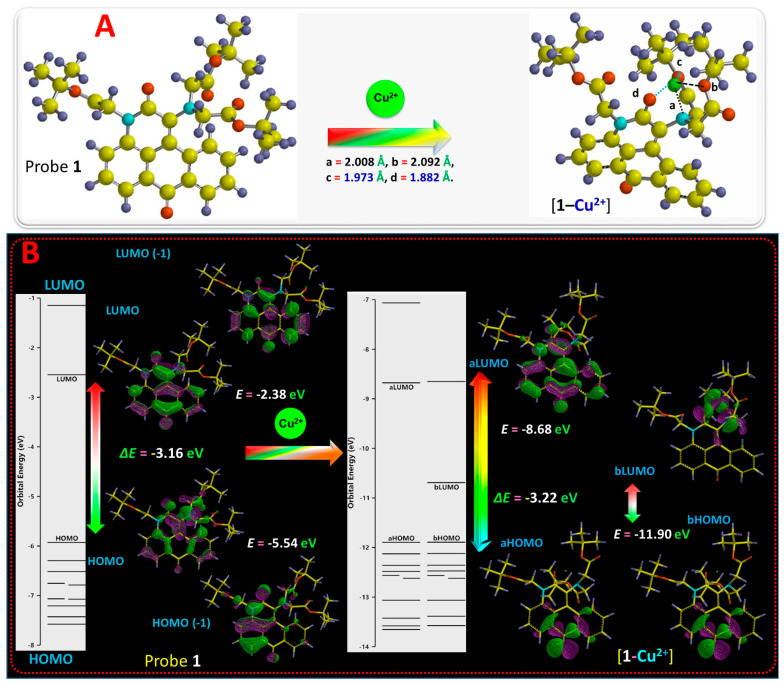
(**A**) Energy-minimized structures of probe **1** and the [**1**-Cu^2+^] complex in a ball and spoke model and (**B**) electronic distributions and energies of their frontier molecular orbitals (FMOs; HOMOs and LUMOs) obtained from DFT calculations at the B3LYP/6-31G* level. Individual atoms are color coded for a clear view: N = sky blue (cyan), O = red, C = yellow, and H = light purple. The letters (a-d) represent individual bond lengths between the metal ion and the chelating atoms.

To further understand the molecular interactions of Cu^2+^ with probe **1**, density functional theory (DFT) calculations were performed at the B3LYP/6-31G* level (Figure 7 and Figures S18–S20). Such calculations provide information about the energy levels and electronic distributions of the frontier molecular orbitals (FMOs) (i.e., HOMO [highest occupied molecular orbital] and LUMO [lowest unoccupied molecular orbital]). As the 1:1 complex between probe **1** and the metal ion was demonstrated by various experiments (fluorescence, UV–Vis, and NMR studies), we used this stoichiometry as a model for the calculations. The calculations indicate that four atoms (i.e., amine N, two O’s of two different ester groups, and carbonyl O of the amide in probe **1**) coordinate with Cu^2+^ to form the tetrahedral complex (Figure 7A and Figures S18–S20). Upon complexation, the Δ*E_H/L_* (HOMO and LUMO energy gap) was increased from 3.16 to 3.22 eV (Figure 7B), qualitatively explaining the blue shifts of the absorption and fluorescence spectra observed for probe **1** upon interaction with Cu^2+^. A minor deviation from the experimental result may be due to the fact that we carried out the calculations under the vacuum condition. The HOMO electron density of probe **1** mainly was distributed around the tertiary amino N and the amide group-containing ring of naphthoquinoline-dione, while the LUMO electron density was localized on the naphthoquinoline-dione ring. The electron densities calculated for the aHOMO and bHOMO of probe **1** complexed with Cu^2+^ were both mainly located around the carbonyl functional group of the naphthoquinoline-dione ring. However, the LUMO electron density of the probe moved to the metal binding site upon complexation with the metal ion (Figure 7B). This electron transfer is most likely responsible for the effective quenching of the green/yellow fluorescence emission of probe **1** upon Cu^2+^ binding (Scheme 2). 

### 2.4. Practical Application

**Figure 8:**
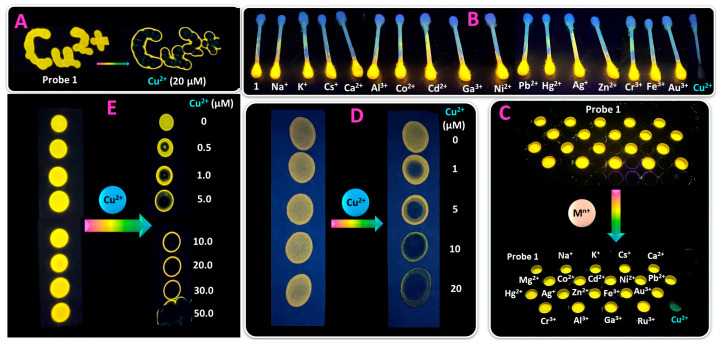
(**A**) Quenching effect of Cu^2+^ on the luminescence of ‘Cu^2+^’ written on the TLC plate using probe **1** (5 µM, ACN) solution. (**B**,**C**) Quenching effect of various metal ions on the luminescence of probe **1**-coated cotton buds (**B**) and silica gels placed in the wells of ELISA plates (**C**). The probe-coated cotton buds and silica gels were prepared by applying the probe **1** solution (5 μM) onto the cotton buds and silica gels. (**D**,**E**) Quenching effect of Cu^2+^ on the luminescent spot of probe **1** (5 μM) on the TLC plate (**D**) and Whatman filter paper (**E**). Different amounts of metal ions were applied to the spots, and the images were obtained under 365 nm illumination.

To examine the practical utility, we first investigated the ability of the probe to detect Cu^2+^ in a solid state. For this purpose, various solid surfaces, such as a TLC plate, cotton, and Whatman filter paper, were used for probe adsorption. The probe adsorbed on these solid supports yielded bright yellow luminescence under 365 nm illumination (Figure 8). The bright yellow ‘Cu^2+^’ written on the TLC plate using the probe **1** solution (5 µM, ACN) was quenched following the application of Cu^2+^ spray (20 µM) (Figure 8A). Similar results were obtained for probe **1** on cotton buds and silica gels (Figure 8B,C). Yellow luminescence of the spots on the filter paper and TLC plate gradually weakened with increasing amounts of Cu^2+^. The filter paper and TLC plate-supported probe **1** showed the ability to detect 0.5 µM and 1 µM Cu^2+^, respectively (Figure 8D,E). 

**Figure 9:**
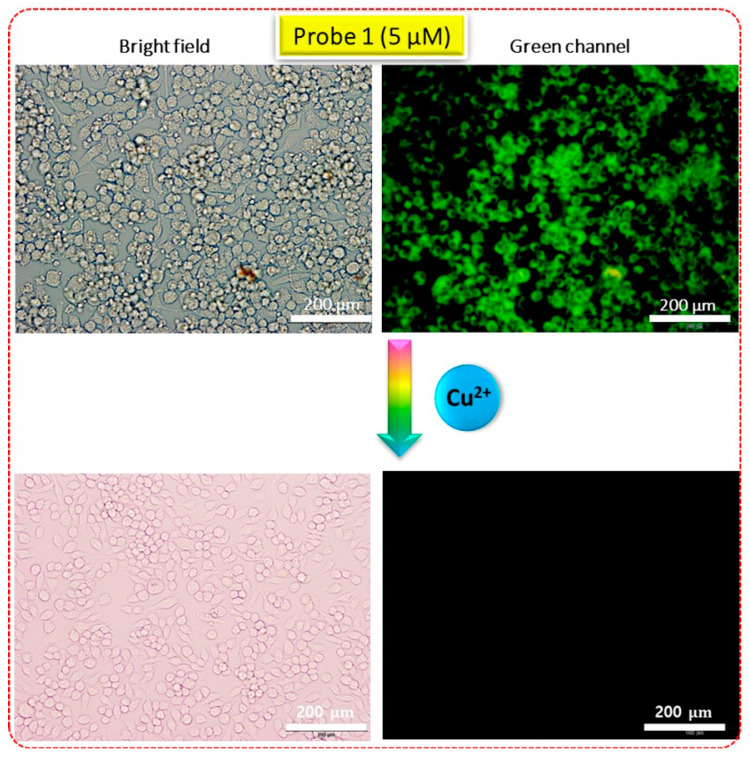
Confocal microscopic images of HeLa cells loaded with probe **1** in the absence (**top**) and presence of Cu^2+^ (20 µM, **bottom**). The HeLa cells were preloaded with probe **1** (5 µM) for 30 min and then treated with Cu^2+^ (20 µM), followed by the acquisition of fluorescence images under excitation at 488 nm. The probe stock solution is prepared using DMSO, and the final DMSO concentration was 0.5% in the sample medium.

Probe **1** was further applied to detect Cu^2+^ in live HeLa cells. For this experiment, HeLa cells were incubated with probe **1** over a range of concentrations (0–20 µM) for 30 min. More than 80% of the cells treated with the probe survived under the conditions, indicating that the probe is only slightly toxic to the cells (Figure S21). Under irradiation at 488 nm, the cells without the probe displayed negligible emission, but incubation of the cells with probe **1** (5 µM) produced an intense green emission in the cytoplasm (Figure 9). This optical change confirms that the probe penetrated the plasma membranes but not the double-layered nuclear membranes. When the HeLa cells containing probe **1** were treated with 20 µM Cu^2+^, the green emission of the cells was completely quenched. This quenching likely originates from the complexation of the probe with metal ions in the cytoplasm. The bright-field images indicate that these cells were viable throughout the experiment. Hence, intracellular Cu^2+^ can be detected in a non-toxic manner using probe **1**, which is crucial for bio-applications.

## 3. Materials and Methods

### 3.1. General Chemicals and Materials

All reagents and solvents used in the experiments, including *t*-butyl bromoacetate, metal perchlorates, tetrabutylammonium bisulfate (TBAHSO_4_), dimethyl sulfoxide (DMSO), THF, acetonitrile (ACN), and potassium carbonate (K_2_CO_3_), were purchased from Aldrich (St. Louis, MO, USA) (analytical grade) and used without further purification. The compound 1-amino-3*H*-naphtho[1,2,3-*de*]quinoline-2,7-dione (compound **3**) was synthesized according to a procedure reported previously [44]. All the tested metal ions were used in the perchlorate forms (M(ClO_4_)_x_), except Ru^3+^ and Au^3+^, which were used in the form of chloride salts. Stock solutions of the metal ions were prepared in ACN (1 mM) or water (10 mM) depending on the sensing medium (ACN or HEPES (pH 7.4: ACN (1:4)) used for metal ion detection. All the fluorescence spectra were recorded on a ChronosBH fluorescence lifetime spectrometer. UV–Vis (ultraviolet–visible) spectra were recorded on a Shimadzu UV-2600PC spectrophotometers with a quartz cuvette (Shimadzu, Kyoto, Japan). The cell holder was maintained at 25 °C. The ^1^H and ^13^C nuclear magnetic resonance (NMR) spectra of all new compounds were recorded on a Bruker 400 MHz spectrophotometer using CDCl_3_ or DMSO-*d*_6_ as a solvent and tetramethylsilane (TMS) as an internal standard. The NMR data are reported as chemical shifts in ppm (parts per million) (δ), multiplicities (s = singlet, d = doublet, m = multiplet), coupling constants (Hz), integration, and interpretation. All spectrophotometric titration curves were fitted with gnu plot software 5.0.

### 3.2. Photo-Physical Studies: Parameters and Conditions

All metal ion-induced color changes, UV–Vis, and fluorescence spectra were obtained in ACN (CH_3_CN) or a binary solvent (HEPES (pH 7.4): ACN (1:4)). All absorption and fluorescence scans were saved as ACSII files and further processed in Excel^TM^ to produce the graphs shown. A stock solution of probe **1** was prepared at 1 mM in ACN and was used for photo-physical studies following appropriate dilution with ACN or a binary solvent (HEPES (pH 7.4): ACN (1:4)). The UV–Vis and fluorescence studies were performed using the probe at 5 and 50 µM, respectively. The titration experiments were carried out by adding Cu^2+^ to the probe **1** solution until the absorption/fluorescence spectra of the solutions reached saturation. The association constants between the probe and Cu^2+^ were determined by fitting the absorption and fluorescence spectral data obtained from the titration experiment of probe **1** with Cu^2+^. The titration data were fitted with the global analysis program gnu plot software.

### 3.3. Theoretical Calculations

Energy-optimized conformations of probe **1** and its Cu^2+^ complex were obtained via gradient-correlated DFT (density functional theory) calculations using Becke’s three-parameter exchange functional [45] and the Lee–Yang–Parr (B3LYP) exchange–correlation function [46] with 6-31G* basis sets for C, H, N, and O. B3LYP/6-31G* calculations were employed for excited-state optimization. For simplicity, all calculations were carried out in a vacuum to avoid the solvent effect. All stationary points were verified as the minima via calculations from Hessian and harmonic frequency analyses [47,48].

### 3.4. Synthesis of Probe ***1***

#### 3.4.1. Tert-butyl 2-(1-amino-2,7-dioxo-2,7-dihydro-3H-cnaphtho[1,2,3-de]quinolin-3-yl)acetate (**2**) 

A solution of 1-amino-3*H*-naphtho[1,2,3-*de*]quinoline-2,7-dione (compound **3**, 1.13 g, 5 mmol) in DMSO (10 mL) containing K_2_CO_3_ (1.38 g, 10 mmol) and TBAHSO_4_ (phase transfer catalyst; 2%) was stirred at room temperature for 20 min. Next, *tert*-butyl bromoacetate (0.975 mg, 5.0 mmol) was added to the reaction mixture, and the resulting solution was stirred at room temperature for 48 h. The progress of the reaction was monitored by TLC; upon reaction completion, a brine solution was added. A solid material collected by filtration was identified as unreacted compound **3**. The filtrate was concentrated and purified by column chromatography to yield the product (**2**) (50%) as a yellow solid. ^1^H NMR (400 MHz, CDCl_3_): δ 1.49 (s, 9H, 3 × CH_3_), 5.13 (s, 2H, CH_2_), 5.89 (s, 2H, NH_2_), 7.35 (d, *J* = 8.0 Hz, 1H, ArH), 7.51–7.57 (m, 2H, 2 × ArH), 7.76 (t, *J* = 8.0 Hz, 1H, ArH), 8.33 (d, *J* = 8.0 Hz, 1H, ArH), 8.51 (d, *J* = 8.0 Hz, 1H, ArH), and 8.54 (d, *J* = 8.0 Hz, 1H, ArH); ^13^C NMR (100 MHz, CDCl_3_): δ 28.24, 46.02, 83.40, 107.65, 117.91, 120.93, 123.85, 125.96, 126.00, 128.11, 128.91, 131.85, 132.62, 133.54, 135.57, 136.77, 159.03, 166.82, and 182.69. HRMS (M + H^+^) = 377.1499; C_22_H_21_N_2_O_4_ (theoretical (M + H^+^) = 377.1501).

#### 3.4.2. Probe **1**

Compound **2** (190 mg, 0.5 mmol) was stirred vigorously in THF (20 mL) at room temperature, followed by the addition of K_2_CO_3_ (1.38 g, 10 mmol) and TBAHSO_4_ (phase transfer catalyst; 2%) for one hour under an N_2_ atmosphere. Then, *t*-butylbromoacetate (1.95 g, 10 mmol) was added dropwise to the reaction mixture, followed by stirring at room temperature for 72 h. TLC was applied to monitor the progress of the reaction; upon reaction completion, a brine solution was added. This solution was extracted with an equal portion of DCM (x2) to extract the organic compounds completely. The solution was dried with anhydrous Na_2_SO_4_, concentrated, and subjected to column chromatography to afford the pure product (**1**) (50%) as a red solid. ^1^H NMR (CDCl_3_, 400 MHz): δ 1.32 (s, 9H, 3 × CH_3_), 1.38 (s, 9H, 3 × CH_3_), 1.45 (s, 9H, 3 × CH_3_), 3.95 (d, *J* = 18.0 Hz, 1H (NCH), one H of rigid two NCH′H), 4.93 (d, *J* = 17.2 Hz, 1H (NCH′), one H of rigid two NCH′H), 5.15 (s, 1H of NCH_2_, the other H of this CH_2_ could exchange with D due to its H-bonding with –C=O (oxygen)), 5.16 (d, *J* = 17.2 Hz, 1H (NCH′), one H of rigid two NCH′H), 5.63 (d, *J* = 18.0 Hz, 1H (NCH), one H of rigid two NCH′H), 7.29 (d, *J* = 8.0 Hz, 1H ArH), 7.53 (t, *J* = 8.0 Hz, 1H ArH), 7.55–7.60 (m, 2H, 2 × ArH), 8.32 (d, *J* = 8.0 Hz, 1H ArH), and 8.37–8.39 (m, 2H, 2 × ArH); ^1^H NMR (DMSO-*d*_6_, 400 MHz): δ 1.25 (s, 9H, 3 × CH_3_), 1.32 (s, 9H, 3 × CH_3_), 1.43 (s, 9H, 3 × CH_3_), 4.22 (d, *J* = 18.0 Hz, 1H (NCH), one H of rigid two NCH′H), 5.12 (q, *J* = 17.6 Hz, 2H, 2 × (NCH′)), 5.28 (d, *J* = 18.0 Hz, 1H (NCH′), one H of rigid two NCH′H), 5.67 (s, 1H of NCH_2_, the other H of this CH_2_ could exchange with D due to its H-bonding with –C=O (oxygen)), 7.64 (t, *J* = 8.0 Hz, 1H ArH), 7.71–7.75 (m, 2H, 3 × ArH), 8.18 (d, *J* = 8.0 Hz, 1H ArH), and 8.25–8.27 (m, 2H, 2 × ArH); ^13^C NMR (CDCl_3_, 100 MHz): δ 28.03, 28.19, 45.60, 56.48, 68.17, 82.11, 82.95, 83.21, 111.17, 117.55, 118.89, 123.57, 126.43, 127.75, 127.93, 128.27, 128.62, 129.32, 131.40, 134.26, 135.03, 157.67, 166.99, 168.93, 169.22, and 182.65. HRMS (M + H^+^) = 605.2861; C_34_H_40_N_2_O_8_ (theoretical (M + H^+^) = 605.2857).

### 3.5. Live Cell Imaging

HeLa cells were obtained from the KCLB (Korean Cell Line Bank, Seoul, Republic of Korea) and grown in MEM (Minimum Essential Media) supplemented with 10% fetal bovine serum (FBS), 100 µg/mL streptomycin, and 100 IU/mL penicillin. The cells were maintained in a humidified incubator at 37 °C at 5% CO_2_. A total of 3.8 × 10^4^ cells were seeded onto a 13 mm glass bottom of a 35 mm confocal dish and grown for one day/24 h (until 60–70% confluence). The probe stock solution was prepared by dissolving probe **1** in DMSO. Experiments were performed in triplicate in FBS and antibiotic-free medium as follows. HeLa cells were incubated with probe **1** (5 µM) at 37 °C at 5% CO_2_ for half an hour. The final DMSO concentration was 0.5% in the sample. After washing the incubated sample twice with 1 x phosphate-buffered saline (PBS) (pH = 7.4), the cells were further incubated with Cu^2+^ (20 µM, H_2_O) for half an hour under the same conditions and were washed three times with 1 × PBS. A K1-Fluo confocal laser scanning microscope with a 40× oil immersion objective lens was used to obtain confocal microscopy images. Bright-field images after treatment with probe **1** and Cu^2+^ were obtained to evaluate the cell viability. 

## 4. Conclusions

We prepared a new naphthoquinoline-dione-based probe **1** containing three ester groups starting from compound **3** with no ester groups. While compound **3** was selective for Hg^2+^ sensing, the new probe **1** exhibited high selectivity for Cu^2+^ in ACN, as well as in a 20% aqueous ACN solution. The probe displayed a color change from yellow to colorless upon selective interaction with Cu^2+^. A ratiometric approach using two absorption values (***A***_413_/***A***_476_) allowed the detection of Cu^2+^ at as low as 1.0 µM. In addition, the yellow fluorescence emission of the probe in the aqueous solution (HEPES (pH 7.4): ACN = 1:4) was selectively quenched in the presence of Cu^2+^ among various metal ions. Hence, probe **1** provides a dual sensing method for Cu^2+^ detection (absorption and fluorescence emission) and is chromo-fluorogenic. Fluorescence titration of the probe with Cu^2+^ produced a low calculated LOD of 5 nM. Fluorescence, UV–visible, and ^1^H-NMR titration studies, in addition to DFT calculations, supported the 1:1 complex formation between Cu^2+^ and probe **1**. This probe showed the ability to detect Cu^2+^ in a real sample (sea/drain water) with an observed LOD of 0.5 µM, which signifies its potential for on-site monitoring of the metal ion. In addition, the probe was successfully used for Cu^2+^ detection after adsorption onto solid supports, such as cotton, filter paper, a TLC plate, and silica gels. Importantly, the probe was able to penetrate cell membranes and detect Cu^2+^ in the cytoplasm of HeLa cells. Therefore, this study provides insight into the development of new chromo-fluorogenic heteroaromatic sensors for the detection of different analytes.

## Data Availability

The data presented in this study are available in article and Supplementary Materials.

## Supplementary Materials

The following supporting information can be downloaded at: https://www.mdpi.com/article/10.3390/molecules29040808/s1.
