# Supplementary material for: A Naphthoquinoline-Dione-Based Cu2+ Sensing Probe with Visible Color Change and Fluorescence Quenching in an Aqueous Organic Solution
Source: Molecules. 2024 Feb 9;29(4):808. doi: 10.3390/molecules29040808 (PMC10891706; doi:10.3390/molecules29040808)
Supplement: Supplementary file 1 [file molecules-29-00808-s001.zip › molecules-2822147-supplementary.pdf]

A Naphthoquinoline-Dione-Based Cu^{2+} Sensing Probe with Visible Color Change and Fluorescence Quenching in an Aqueous Organic Solution

Ashwani Kumar* and Pil Seok Chae*

Department of Bionano Engineering, Hanyang University, Ansan, 15588, Republic of Korea

Corresponding author Tel.: +82-31-400-5205

E-mail addresses: ashwanirubal@gmail.com, ashwani@hanyang.ac.kr, pchae@hanyang.ac.kr

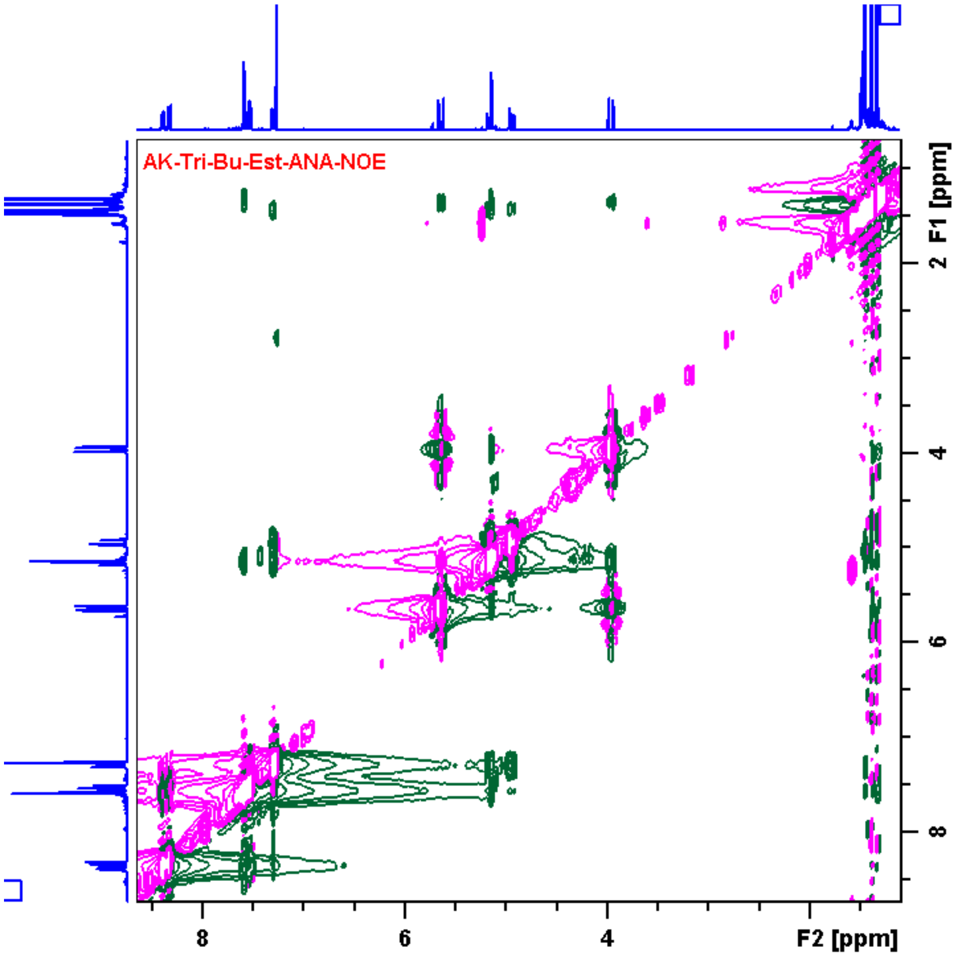

Figure S1. ^1H-^1H NOESY NMR spectrum of probe **1** ($CDCl_3$).

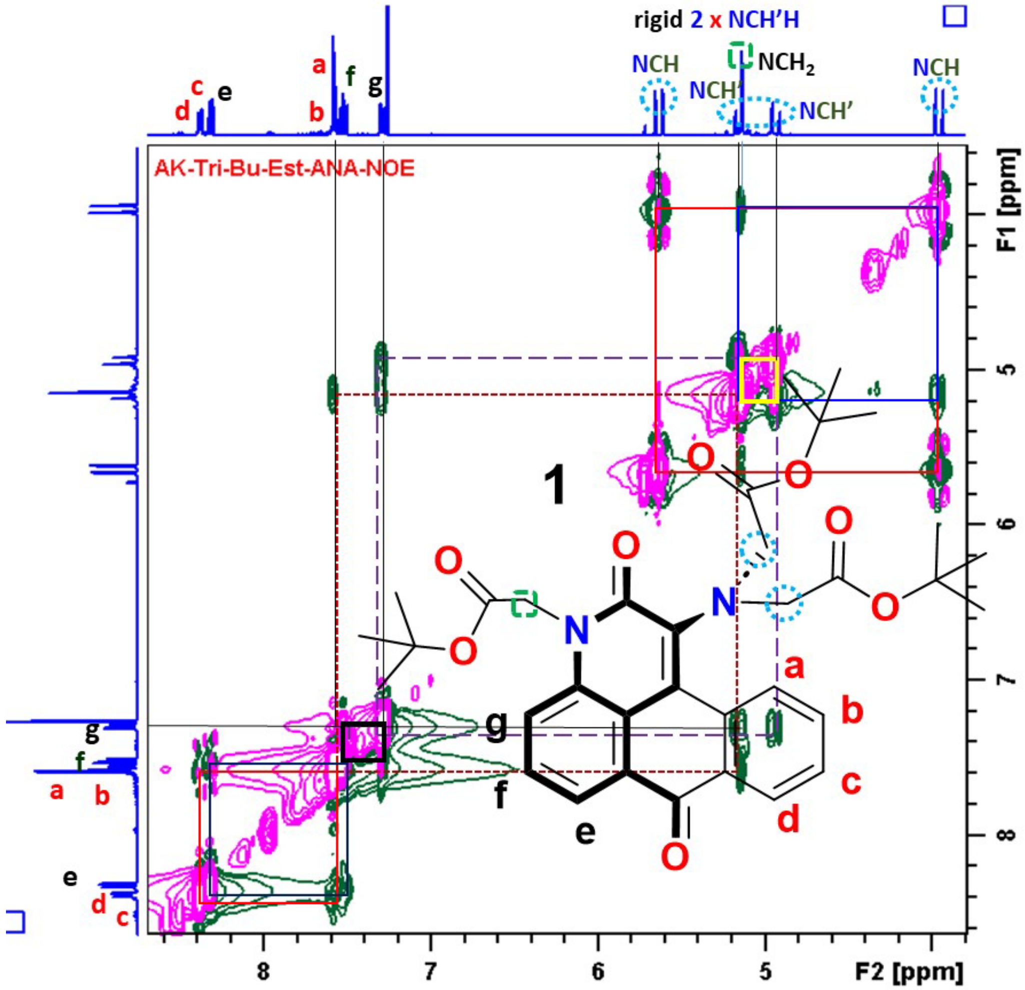

Figure S2. Analysis of ^{1}H-^{1}H NOESY NMR spectrum of probe **1** ($CDCl_3$).

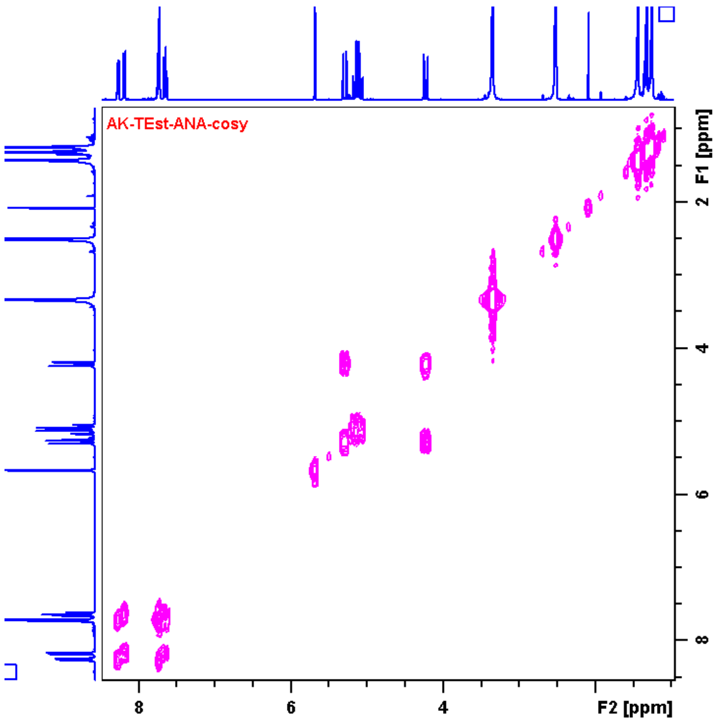

Figure S3. ^{1}H-^{1}H COSY NMR spectrum of probe **1** in DMSO-d$_6$.

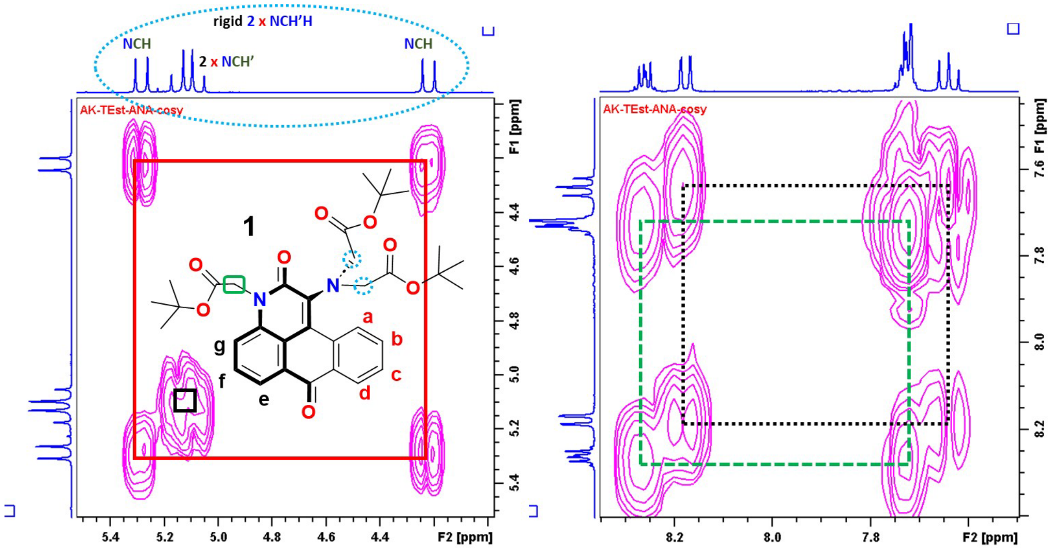

Figure S4. Analysis of ^{1}H-^{1}H COSY NMR spectrum of probe **1** in DMSO-d$_6$.

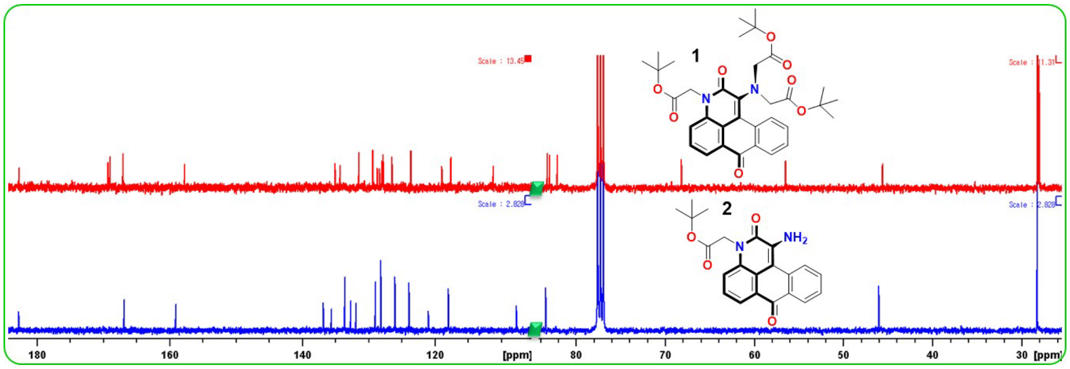

Figure S5. ^{13}C-NMR spectra of probe **1** and compound **2** in $CDCl_3$.

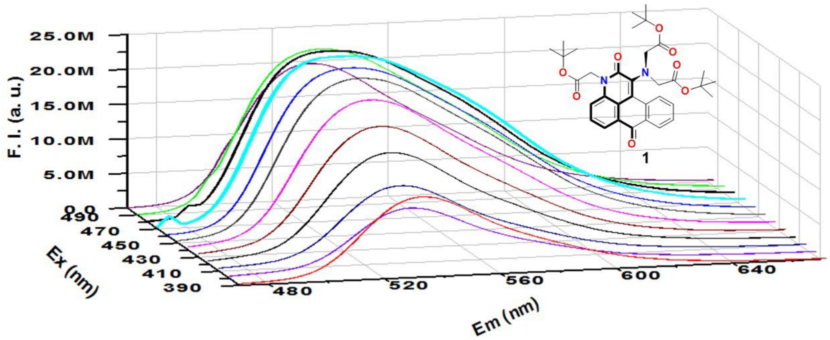

Figure S6. Fluorescence spectra of probe **1** (10 μM, CH_3CN) when excited in a range excitation wavelength from 390 to 500 nm. Excitation at 470 nm (cyan) showed the maximum emission/ quantum yield.

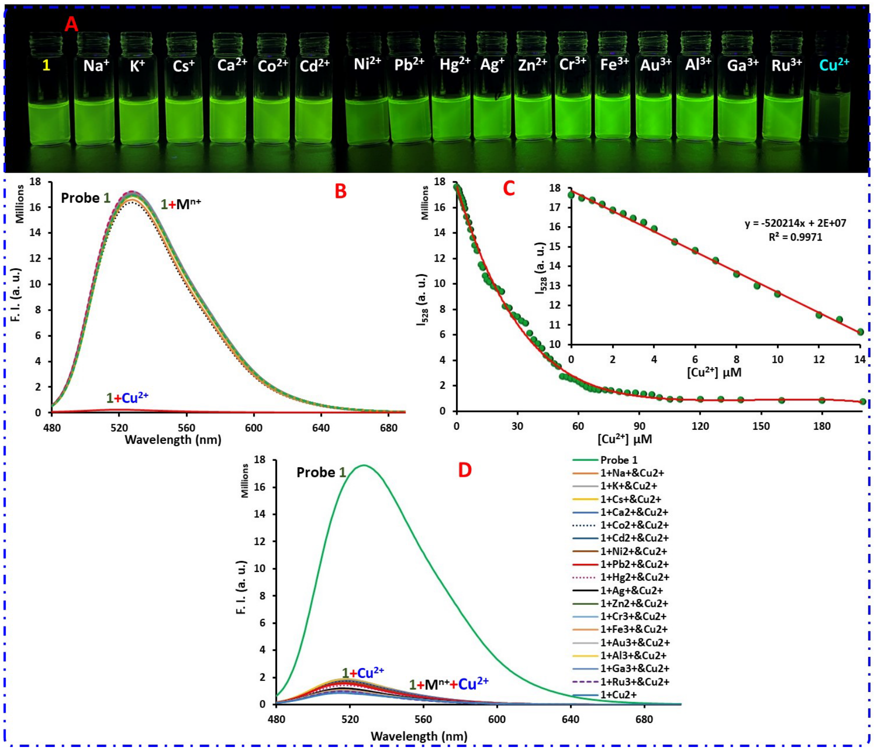

Figure S7. Fluorescence study of probe **1** solutions (5 µM, ACN) upon addition of various metal ions including Cu^{2+}. (A) Luminescence image and (B) fluorescence spectra of probe **1** solution with the different metal ions. (C) Fitting of the fluorescence titration data of probe **1** (5 µM, ACN) with Cu^{2+} ($\boldsymbol{I_{528}}$ vs [Cu^{2+}]). The inset shows the linear relationship at low [Cu^{2+}] concentration. Points and line represent experimental values and curve fit, respectively. (D) Fluorescence interference of various metal ions (100 µM each) for the Cu^{2+} (70 µM) detection of probe **1** (5 µM, ACN), λ_{ex} = 470 nm, Ex. Slit width = 2 mm, Em. Slit width = 3 mm.

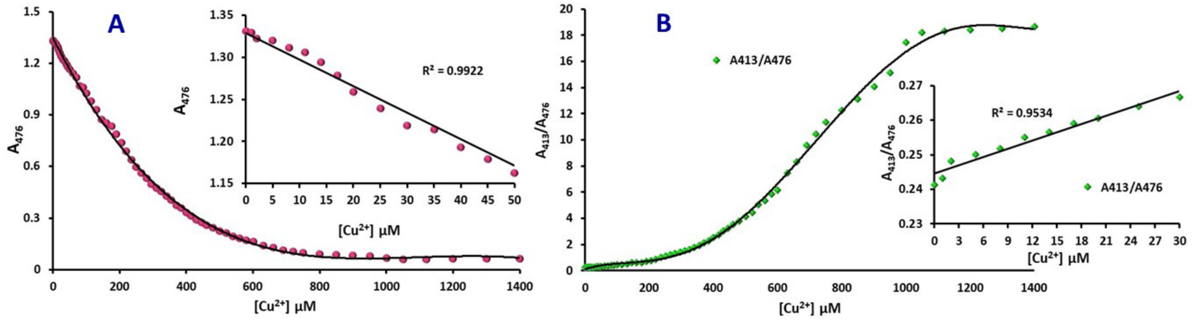

Figure S8. (A) Fitting of the UV-Vis. titration data of probe **1** (50 µM, ACN) with Cu^{2+} ($\boldsymbol{A_{476}}$) vs [Cu^{2+}]). (B) Ratio-metric changes of the absorbance ratio ($\mathbf{A_{413}/A_{476}}$) as a function of [Cu^{2+}]. The inset shows the linear relationship at low concentration range of Cu^{2+}. Points and line represent experimental values and non-linear fitting curve. The inset shows a linear fitting in low concentration range of Cu^{2+}.

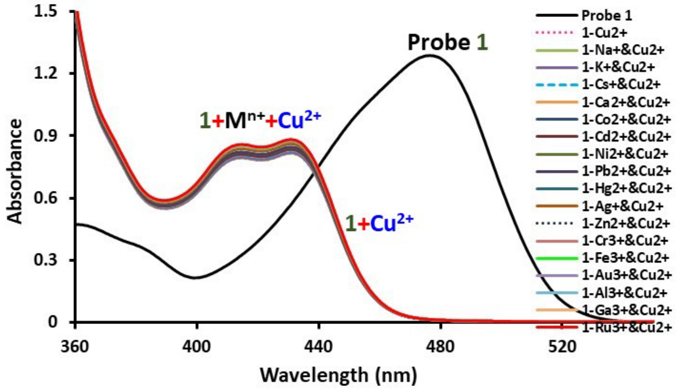

Figure S9. UV-Vis. interference of various metal ions (10 equiv. each) for Cu^{2+} (10 eq.) detection of probe **1** (50 µM, ACN).

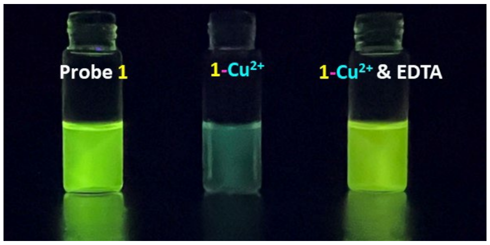

Figure S10. Fluorescence reversibility studies for Cu^{2+} binding of probe **1** (5 µM, ACN). Upon addition of EDTA (disodium ethylene diamine tetra-acetic acid), green luminescence was completely recovered, indicating the dissociation of Cu^{2+} from the probe (reversibility).

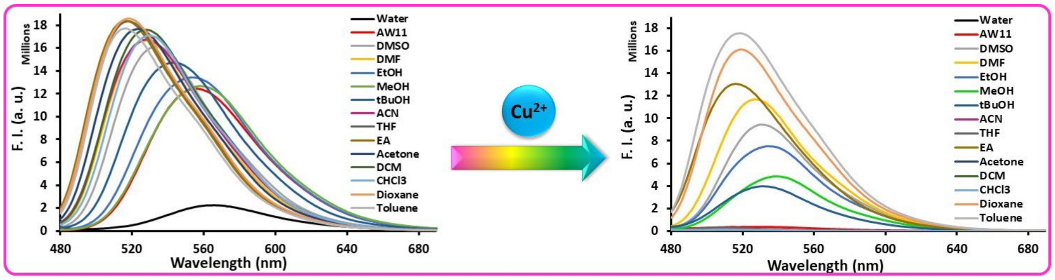

Figure S11. Fluorescence spectral changes of probe **1** (5 µM) in various common solvents upon addition of Cu^{2+} (20 equiv.), λ_{ex} = 470 nm.

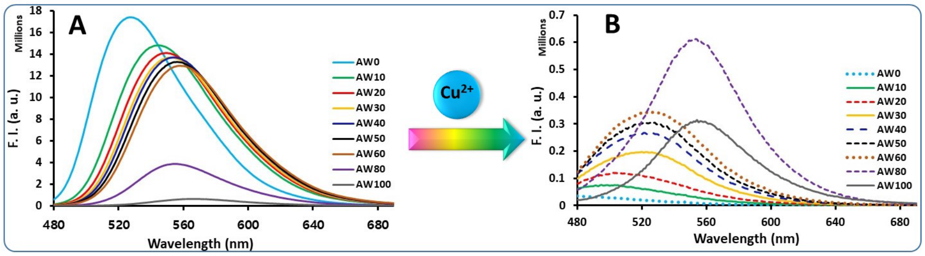

Figure S12. Fluorescence spectral changes of probe **1** (5 µM) in different ratios of ACN and water (binary mixture) in the absence (A) or presence of Cu^{2+} (20 equiv.) (B). A, N, the numbers in the

line designation stand for can, water, and water percentage of water in ACN (CH_3CN), respectively, λ_{ex} = 470 nm.

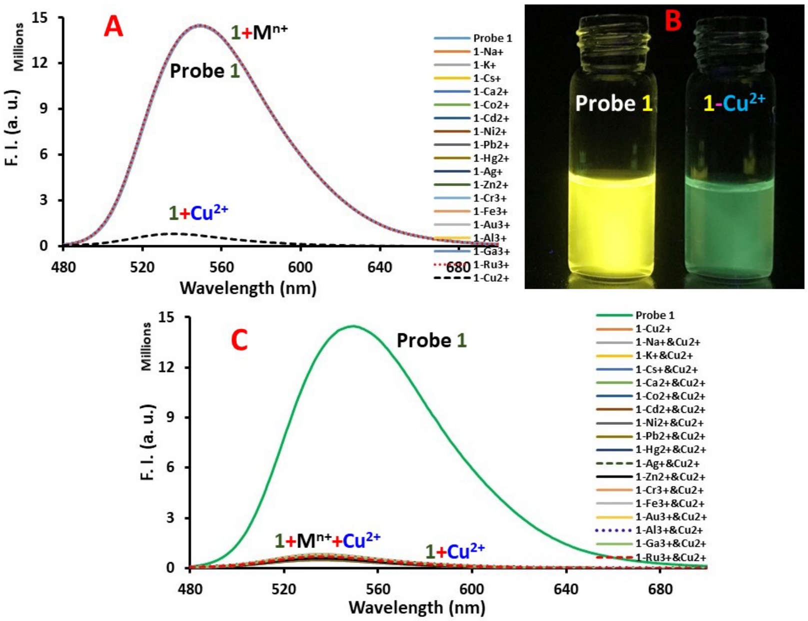

Figure S13. (A) Fluorescence spectra of probe **1** (5 µM, HEPES (pH 7.4): ACN (1:4)) upon additions of various metal ions. (B) Image of the probe solutions before and after addition of Cu^{2+} under 365 nm irradiation. (C) Fluorescence interference of different metal ions (100 µM each) for Cu^{2+} (100 µM) detection of probe **1**. λ_{ex} = 470 nm, Ex. Slit width = 2 mm, Em. Slit width = 3 mm.

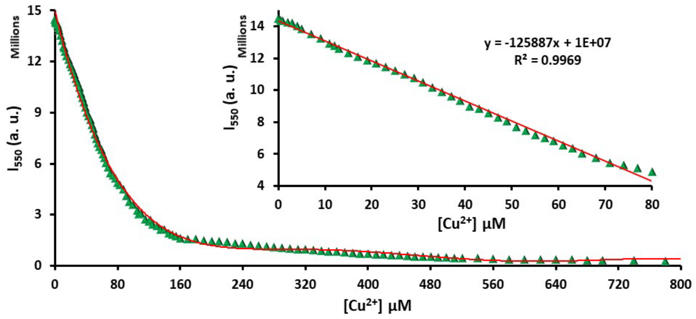

Figure S14. Fitting of the titration data of probe **1** (5 µM, HEPES (pH 7.4): ACN (1:4)) with Cu^{2+}. Fluorescence intensity of the probe at 550 nm (I_{550}) was used for this plot, λ_{ex} = 470 nm. Points and line represent experimental values and non-linear fitting curve. The inset shows a linear fitting in low concentration range of Cu^{2+}.

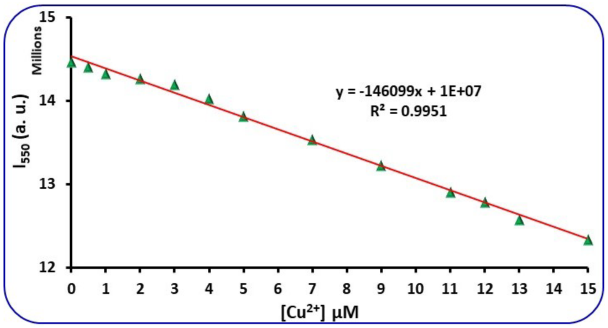

Figure S15. Linear plot between the fluorescence intensity ($\boldsymbol{I}_{550}$) of probe **1** (5 µM, HEPES (pH 7.4): ACN = 1:4) and [Cu^{2+}]. The probe was excited at 470 nm (λ_{ex}) and the resulting fluorescence intensity was measured at 550 nm ($\boldsymbol{I}_{550}$).

*Determination of a calculated LOD for Cu^{2+} sensing of probe **1**.*

An LOD of the probe was calculated from the fluorescence titration data. The fluorescence spectra of the probe were measured three times, and the standard deviation (σ) of the blank measurement was obtained. The slope (*K*) was obtained from the linear plot of the fluorescence intensity of the probe at 550 nm ($\boldsymbol{I}_{550}$) as a function of [Cu^{2+}].

LOD calculation for Cu^{2+} detection

$$\text{LOD} = 3 \times \sigma/K$$

$$\sigma = 0.2498,\ K = 146099 \times 10^3$$

Detection Limit (LOD) = $3 \times 0.2498/146099 \times 10^3 = 5.12 \times 10^{-9}$ M = 5.12 *nM*

Table S1. LOD comparison of probe **1** with other previously reported probes for Cu^{2+} detection.

S. No.	Probe structure	Detection limit in solution (LOD(Flu.))	Solution, selectivity	Reference
**1.**	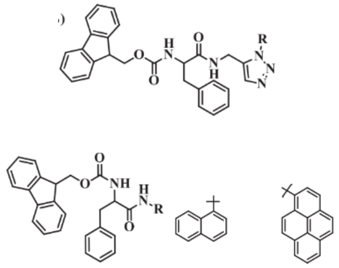	1.669 µM	DMSO<br>Cu^{2+} & Fe^{3+}	J. Photochem. Photobio. A Chem. 442 (2023) 114796
**2.**	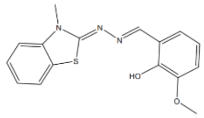	0.37 µM	ACN	J. Chem. Sci. (2022) 134, 43
**3.**	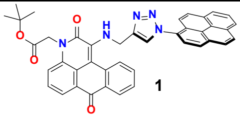	0.2 µM	EtOH	Dyes Pigments 174 (2020) 108092
**4.**	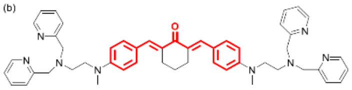	0.379 µM	MeOH/HEPES, pH 7 (1:1)<br>Cu^{2+} selective<br>Respond to Ni^{2+}, Co^{2+}, Zn^{2+}, Cd^{2+}	Dyes Pigments 197 (2022) 109845
**5.**	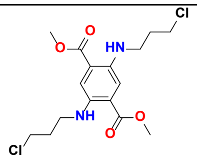	0.303 µM	CH_3CN-H_2O (9:1)<br>Cu^{2+} and<br>TFA & TEA	Dyes Pigments 197 (2022) 109903
**6.**	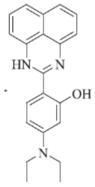	(Not given)	CH_3CN-H_2O (9:1)<br>Cu^{2+} and<br>Al^{3+}	Spectrochim. Acta Part A Molecular and Biomolecular Spectroscopy 293 (2023) 122471
**7.**	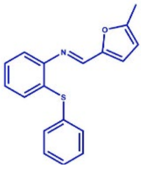	0.194 µM	CH_3CN-H_2O (7:3)	J. Photochem. Photobio. A Chem. 441 (2023) 114750

**8.**	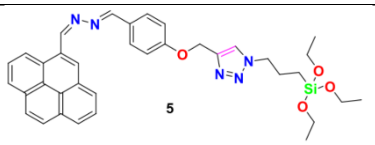	6.33 μM	MeOH	Spectrochim. Acta Part A Mol. Bio.r Spec. 295 (2023) 122618
**9.**	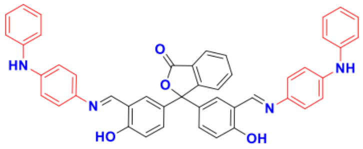	84 nM	CH_3CN:HEPES (1:1, v/v)	J. Photochem. Photobio. A Chem. 437 (2023) 114460
**10.**	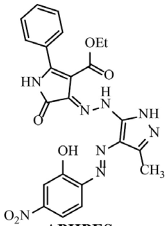	0.59–0.98 μM	EtOH:Tris HCl buffer (9:1)<br>Cu^{2+}, Zn^{2+}, Co^{2+}, only colorimetric	Dyes Pigments 196 (2021) 109795
**11.**	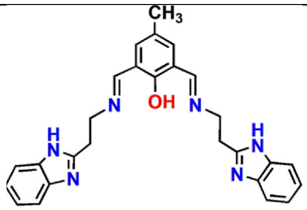	24.4 nM	CH_3CN:HEPES (1:1)<br>Cu^{2+}, Zn^{2+}, CN^-, $P_2O_7^{4-}$, DNA, sensing	Sensors & Actuators: B. Chemical 337 (2021) 129785
**12.**	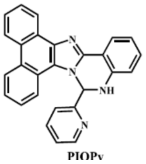	0.1 μM	water/EtOH (1:1, HEPES) pH 7.4	Dyes Pigments 173 (2020) 107916
**13.**	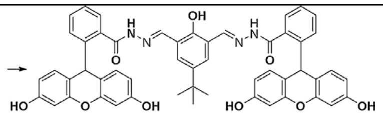	0.21 μM	CH_3CN-H_2O (3:7) pH 7.0 (HEPES)	Sens. Actuators B 282 (2019) 730–742
**14.**	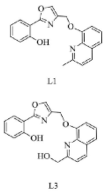	8.2×10^{-8} M	DMF-H_2O (1:1) Tris-HCl buffer<br>pH 7.3	Dyes Pigments 161 (2019) 331-340.
**15.**	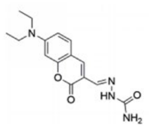	2×10^{-8} M	EtOH-H_2O (1:1)	Sens. Actuators B 288 (2019) 27–37

**16.**	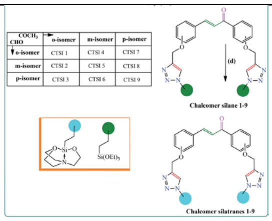	10 μM	MeOH: H_2O (8: 2)	RSC Adv. 5 (2015) 12644–12654
**17.**	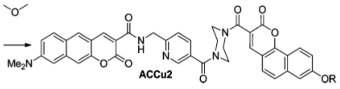	0.84 μM	EtOH/HEPES (9/1, v/v, pH 7.0)	Anal. Chem. 86 (2014) 5353—5359
**18.**	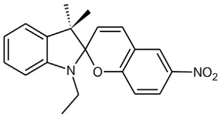	10^{-12} M	DMF/H_2O (9/1, v/v)	Anal. Chim. Acta 1061 (2019) 161-168
**19.**	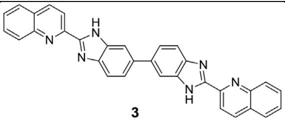 3	1.12 nM (calc.) 0.05 μM (obs.)	DMSO/H_2O, (9/1)	Dyes Pigments 170 (2019) 107651
**20.**	**ZnS** microhybrid particles constructed from interfacial coordination bridge of thiocyanate	1 μM	THT	Dyes and Pigments 172 (2020) 107831
**21.**	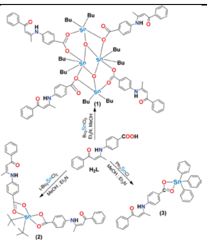	1.47 μM	MeOH	New J. Chem. 43 (2019) 16050--16057
**22.**	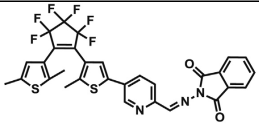	4.1×10^{-8} M	MeOH	Dyes Pigments 161 (2019) 34–43
**23.**	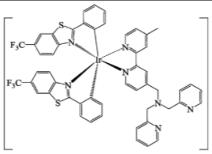 [(CF₃-bt)₂Ir(bp-DPA)]PF₆	0.5 μM	MeOH	Dyes Pigments 161 (2019) 252–260
**24.**	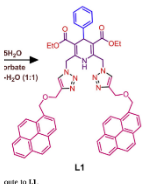	0.53 μM	CH_3CN	Dyes and Pigments 161 (2019) 162–171

**25.**	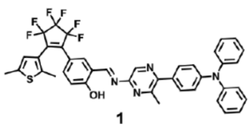 1	2.0 μM	THF	Dye Pigments 173 (2020) 107914.
**26.**	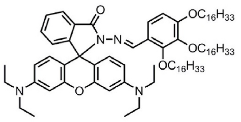	0.829 nM.	Cyclohexanol	Dyes Pigments 192 (2021) 109436
**27.**	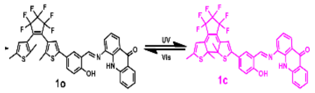	0.395 μM	DMSO<br>Arginine, Cu^{2+}<br>only colorimetric	Dyes Pigments 195 (2021) 109752
**28.**	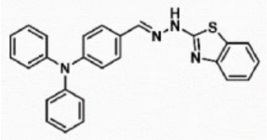	0.45 μM	CH_3CN<br>Cu^{2+}, Fe^{3+}, Zn^{2+}	Dyes Pigments 213 (2023) 111180
**29.**	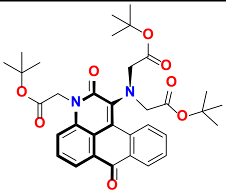 1	*0.5 μM (obs.)*<br>*5.12 nM (calc.)*	*HEPES:ACN (1:4)*	*Present work*

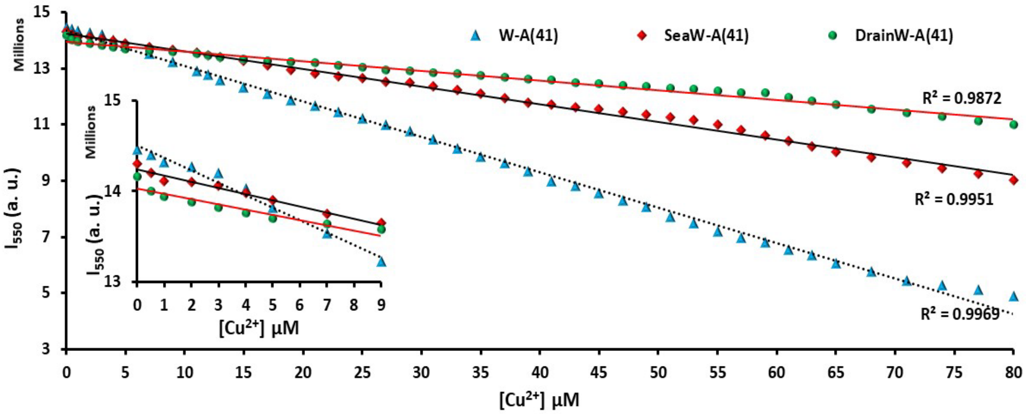

Figure S16. Plot of the fluorescence intensity of probe **1** (5 μM) at 550 nm (***I**$_{550}$*) as a function of [Cu^{2+}] in three different media (tap water (W), sea water (SeaW), and drain water from a lake (DrainW)). The data were obtained from titration experiments of the probe with Cu^{2+}. Points and lines are the experimental values and a linear relationship fit, respectively. The Inset shows magnification of linear relationship showing a limit of detection (LOD) of 0.5 μM for Cu^{2+} sensing in all three cases.

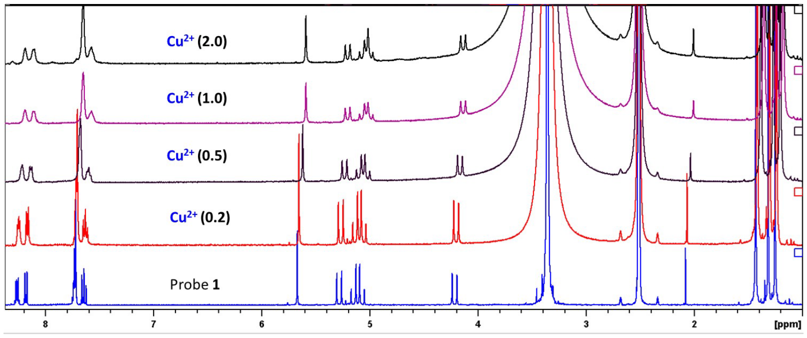

Figure S17. ^{1}H-NMR of titration of probe **1** with $Cu^{2+}(ClO_4)_2.6H_2O$ (DMSO-d_6).

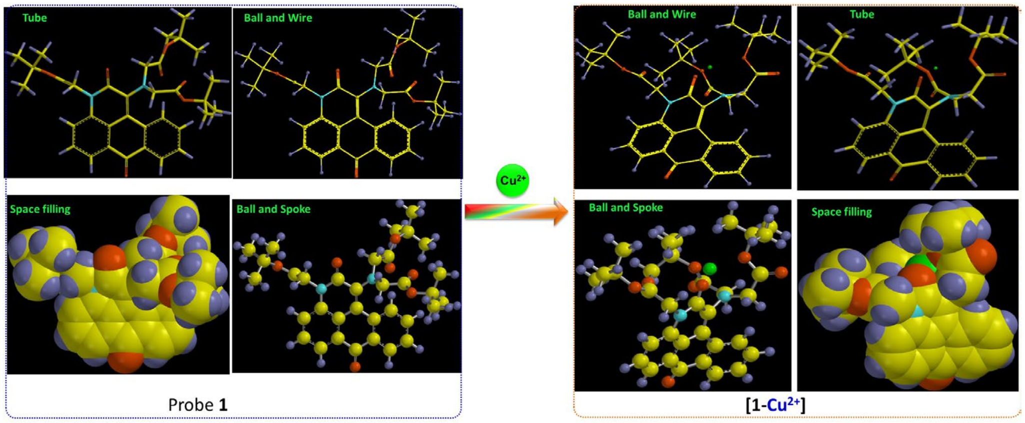

Figure S18. Energy-minimized model structures of probe **1** and its complex with Cu^{2+} obtained from DFT calculations at the B3LYP/6-31G* level; color coding: C = yellow, N = sky blue (cyan), O = red, Cu^{2+} = green, and H = light purple.

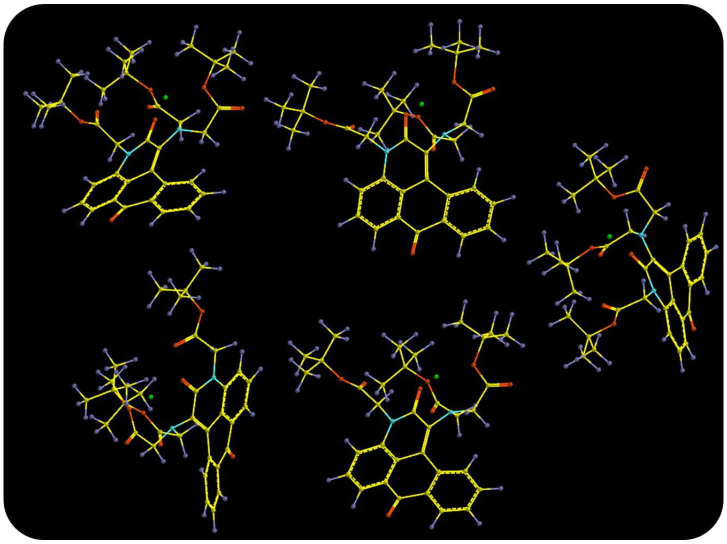

Figure S19. Energy-minimized structures of [probe **1**-Cu^{2+}] at different angle views obtained from DFT calculations at the B3LYP/6-31G* level; color coding: C = yellow, N = sky blue (cyan), O = red, Cu^{2+} = green, and H = light purple.

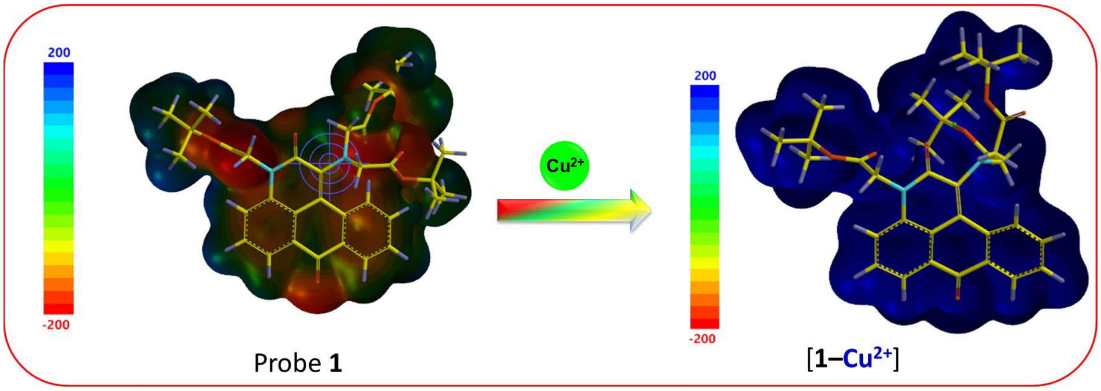

Figure S20. Electrostatic potential map of probe **1** and [**1**-Cu^{2+}] obtained from DFT calculations at the B3LYP/6-31G* level; color coding: C = yellow, N = sky blue (cyan), O = red, Cu^{2+} = green, and H = light purple.

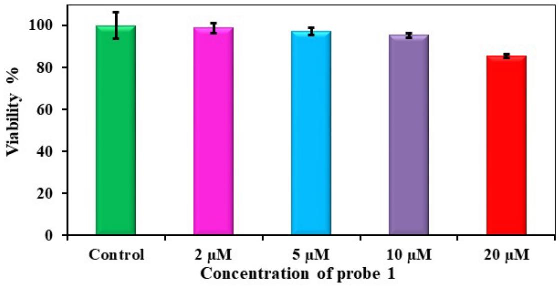

Figure S21. Viability of HeLa Cells incubated with probe **1** at a range of concentrations (0-20 µM).

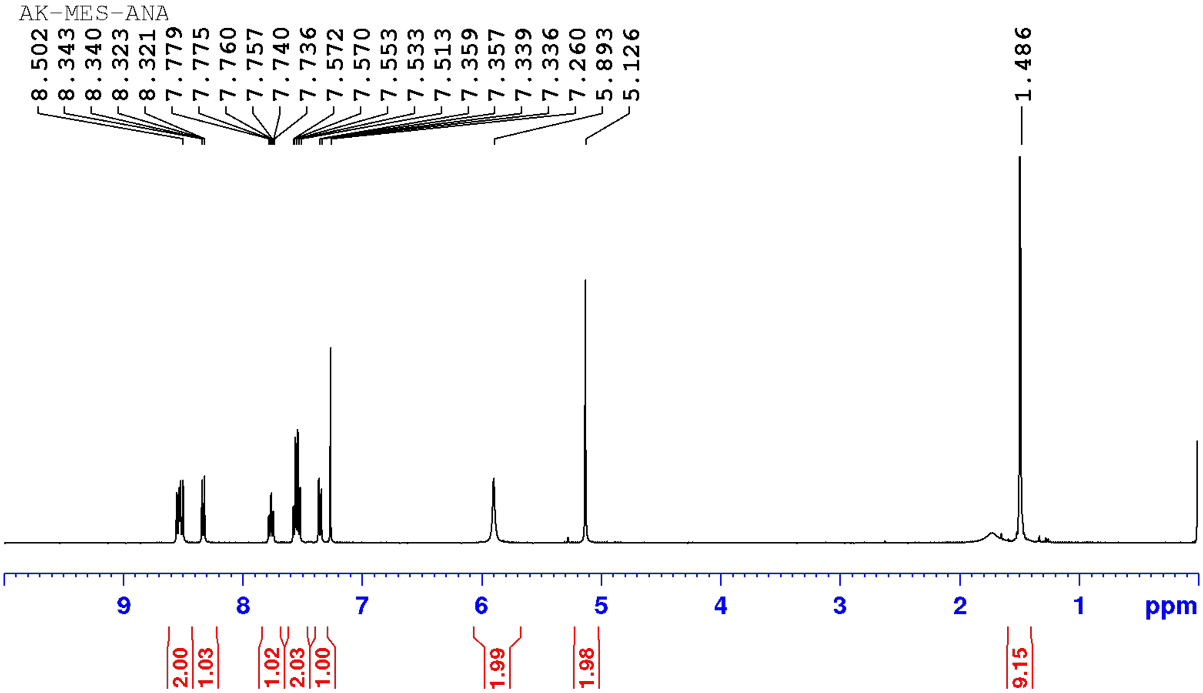

^{1}H-NMR spectrum of probe **2** in $CDCl_3$ (full)

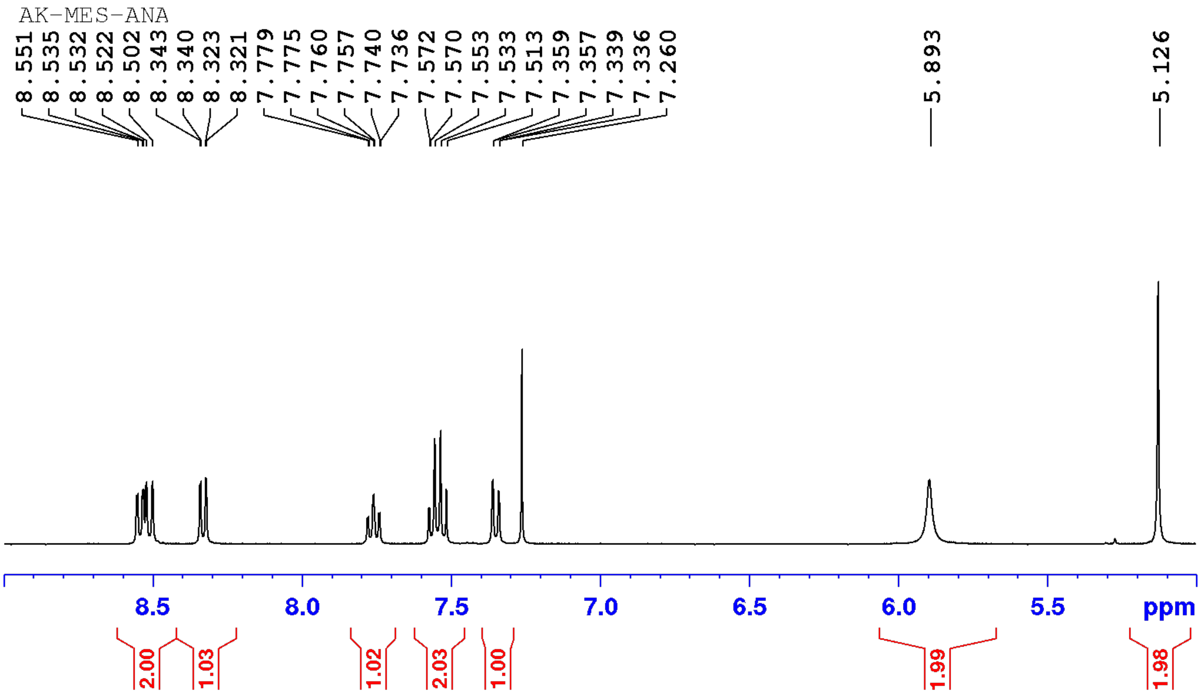

^{1}H-NMR spectrum of probe **2** in $CDCl_3$ (partial)

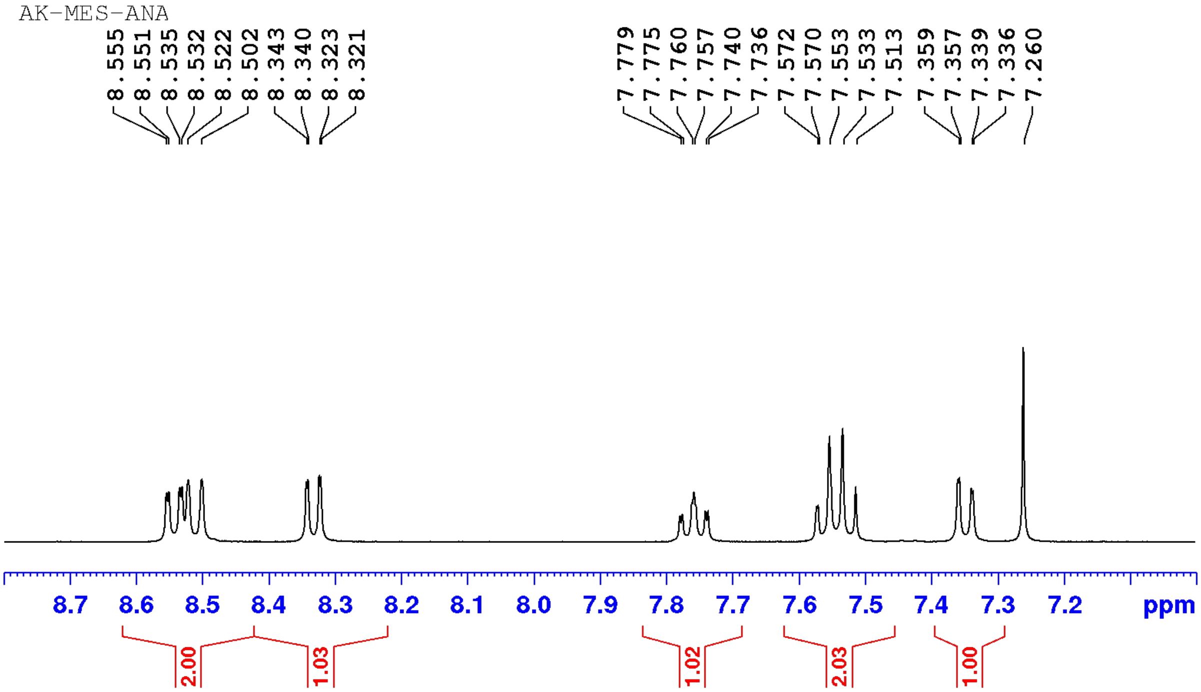

^{1}H-NMR spectrum of probe **2** in $CDCl_3$ (aromatic region)

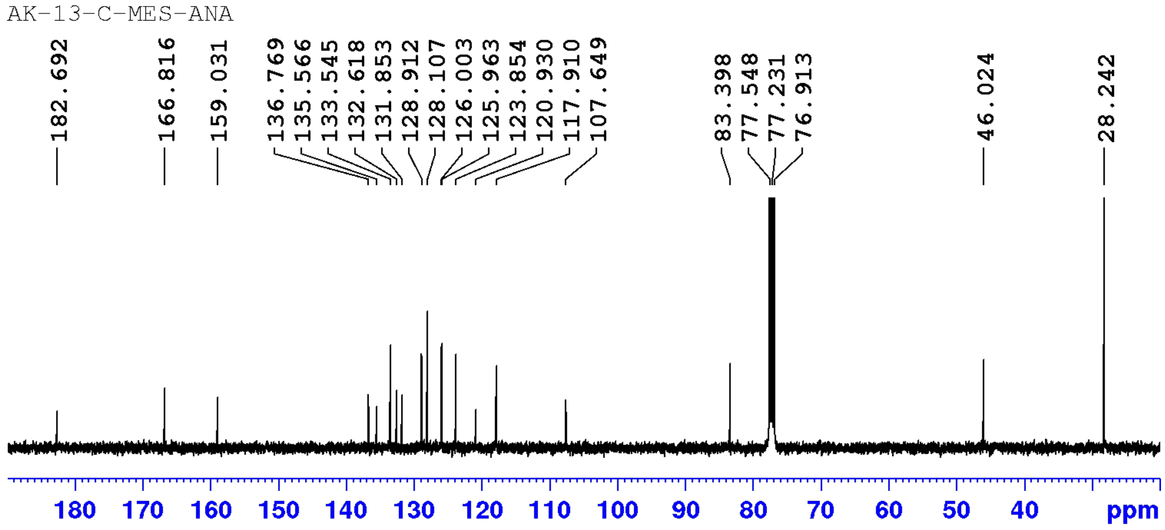

^{13}C-NMR spectrum of probe **2** in $CDCl_3$ (full)

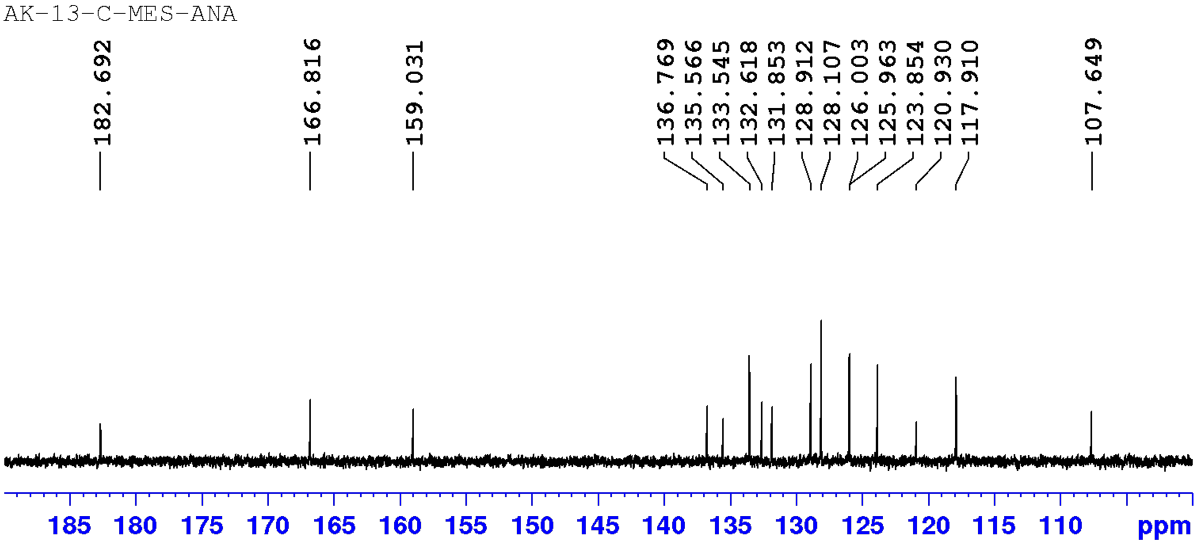

^{13}C-NMR spectrum of probe **2** in $CDCl_3$ (partial)

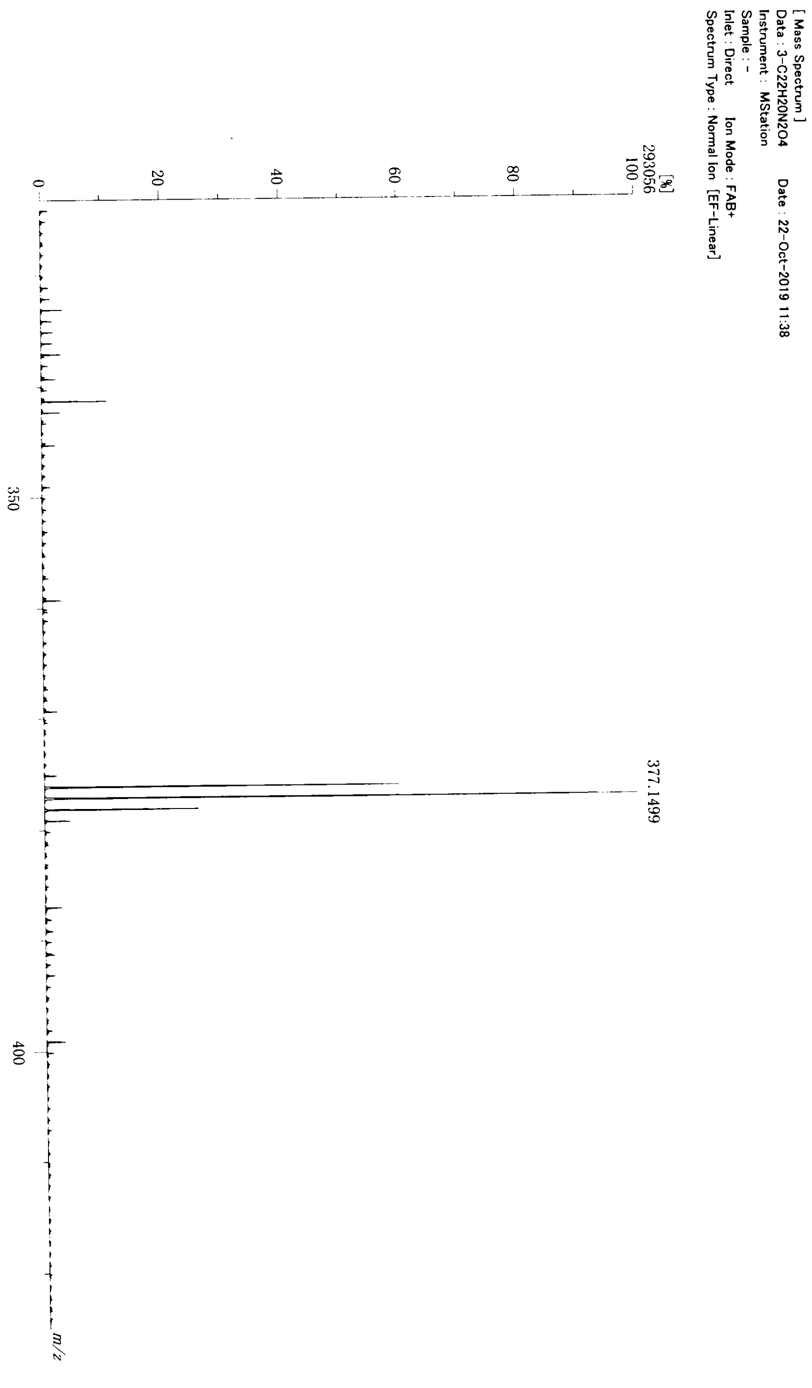

HRMS of compound **2**

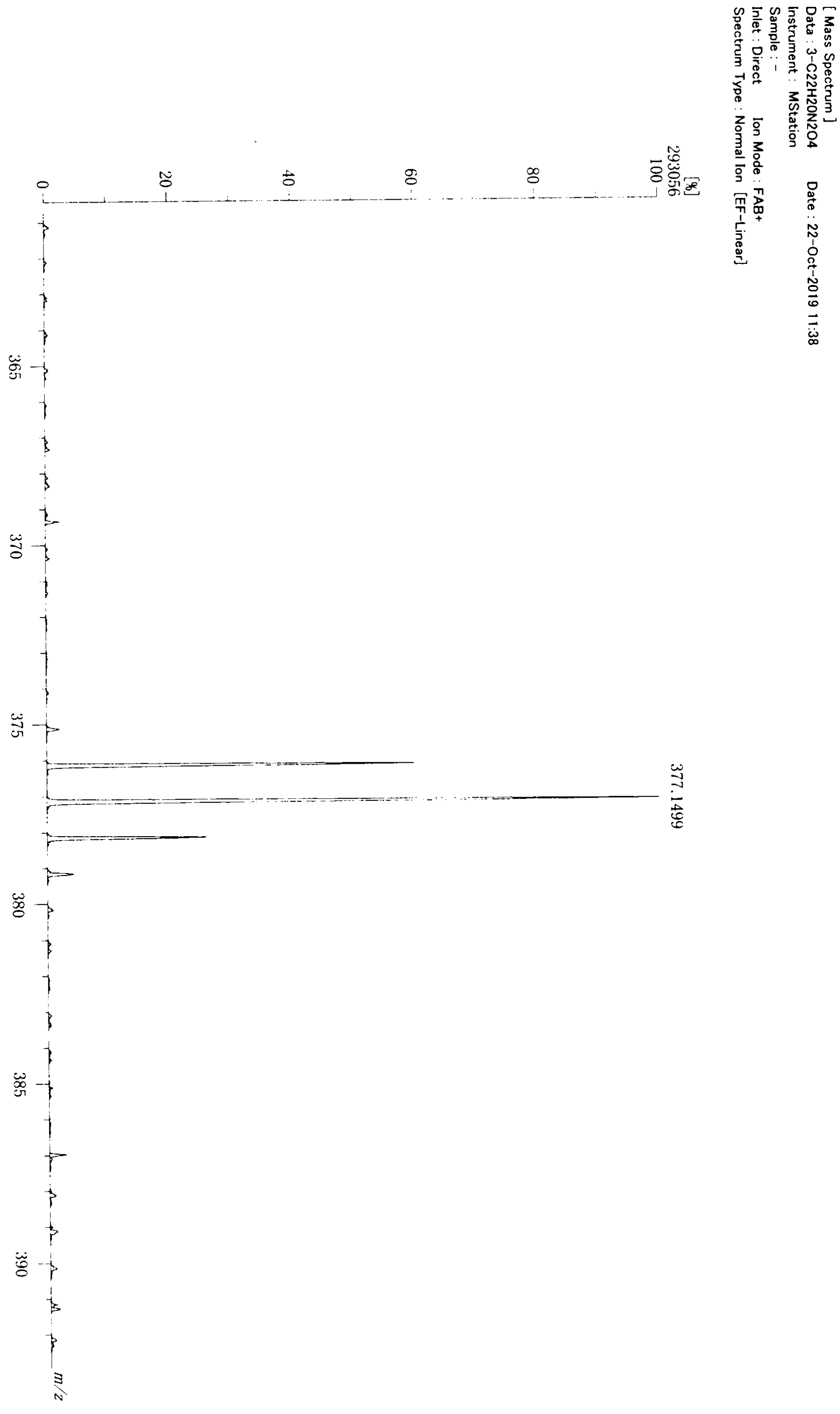

HRMS of compound **2**

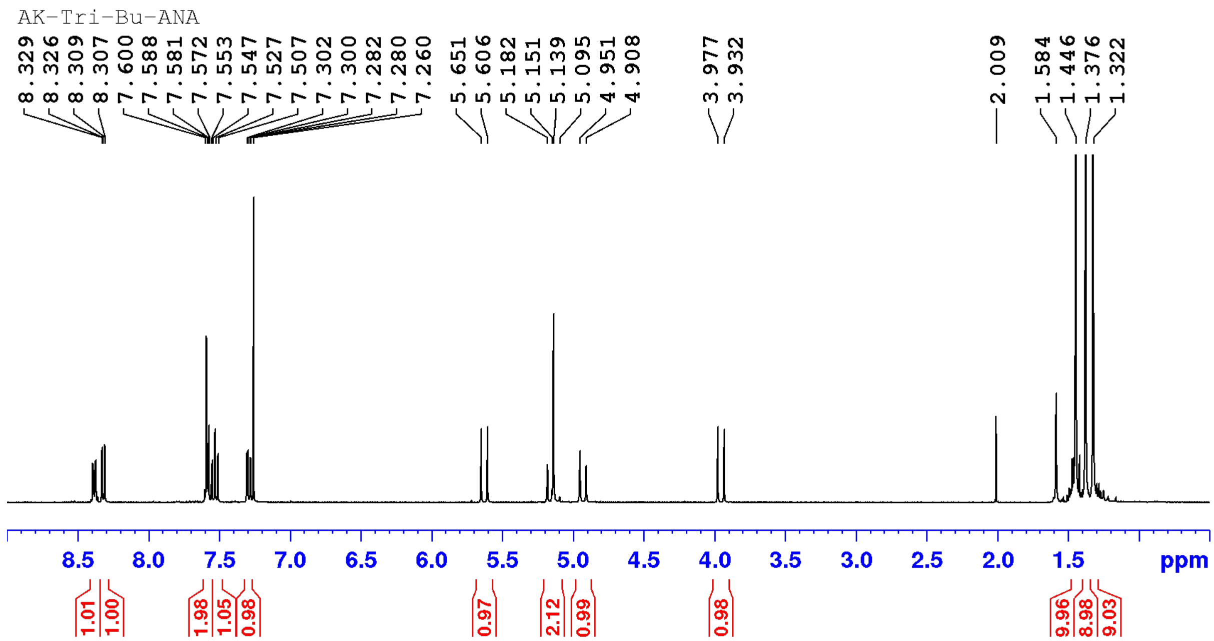

^{1}H-NMR spectrum of probe **1** in $CDCl_3$ (full)

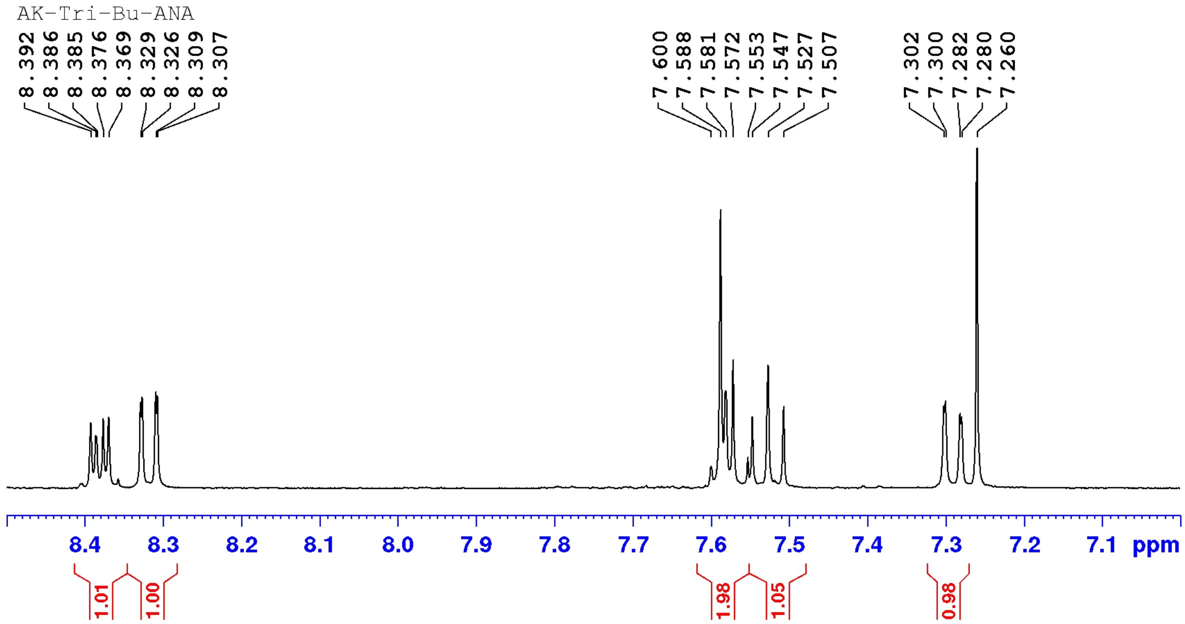

^{1}H-NMR spectrum of probe **1** in $CDCl_3$ (aromatic region)

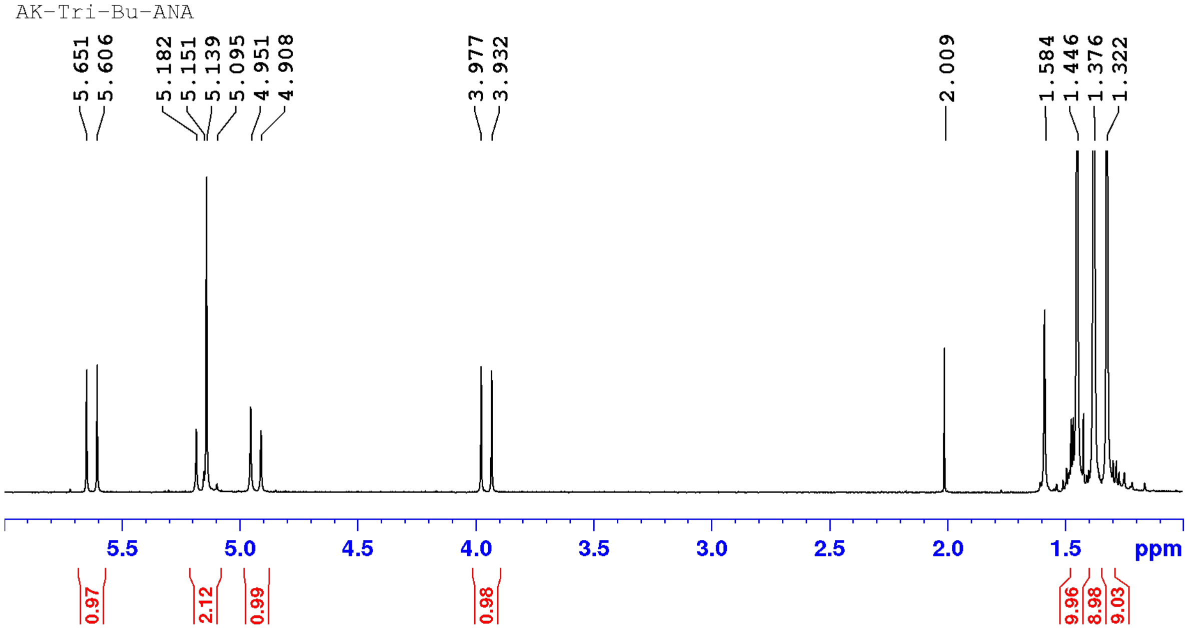

^{1}H-NMR spectrum of probe **1** in $CDCl_3$ (aliphatic region)

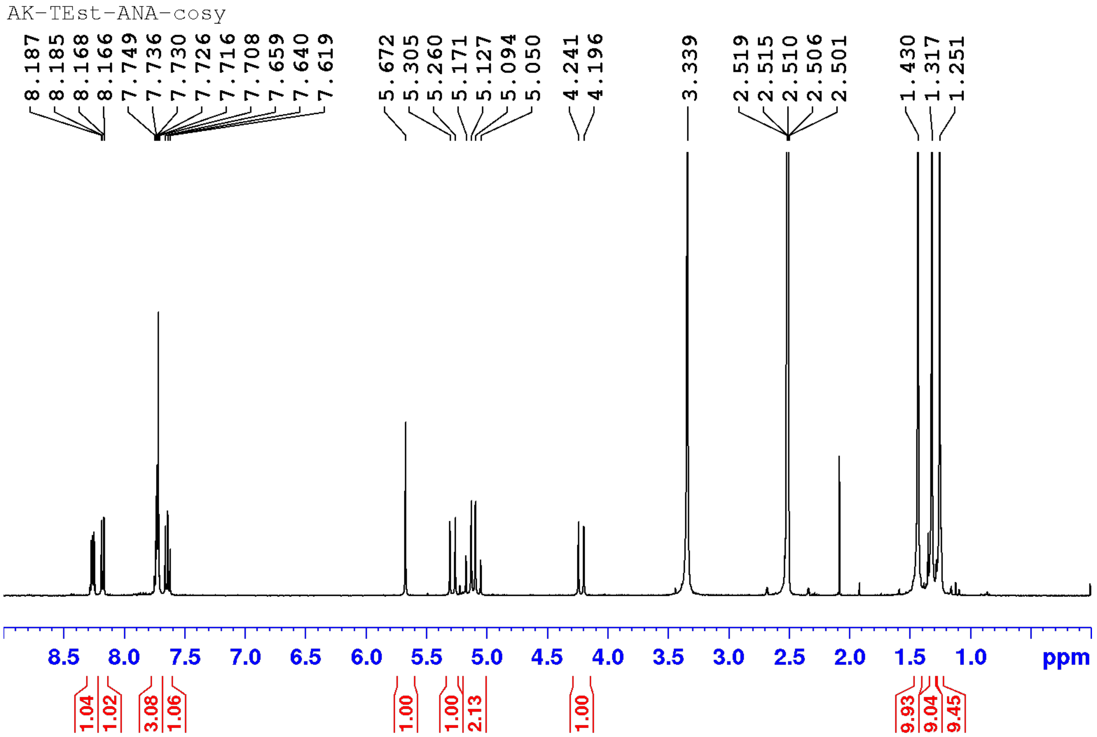

^{1}H-NMR spectrum of probe **1** in DMSO-d_6 (full)

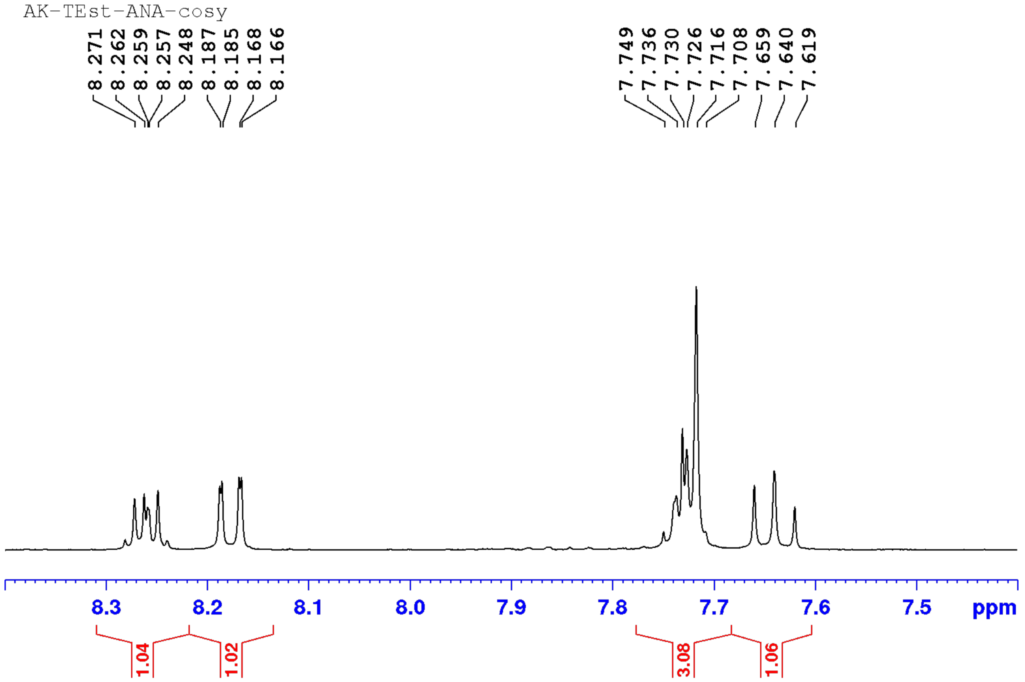

^{1}H-NMR spectrum of probe **1** in DMSO-d_6 (aromatic region)

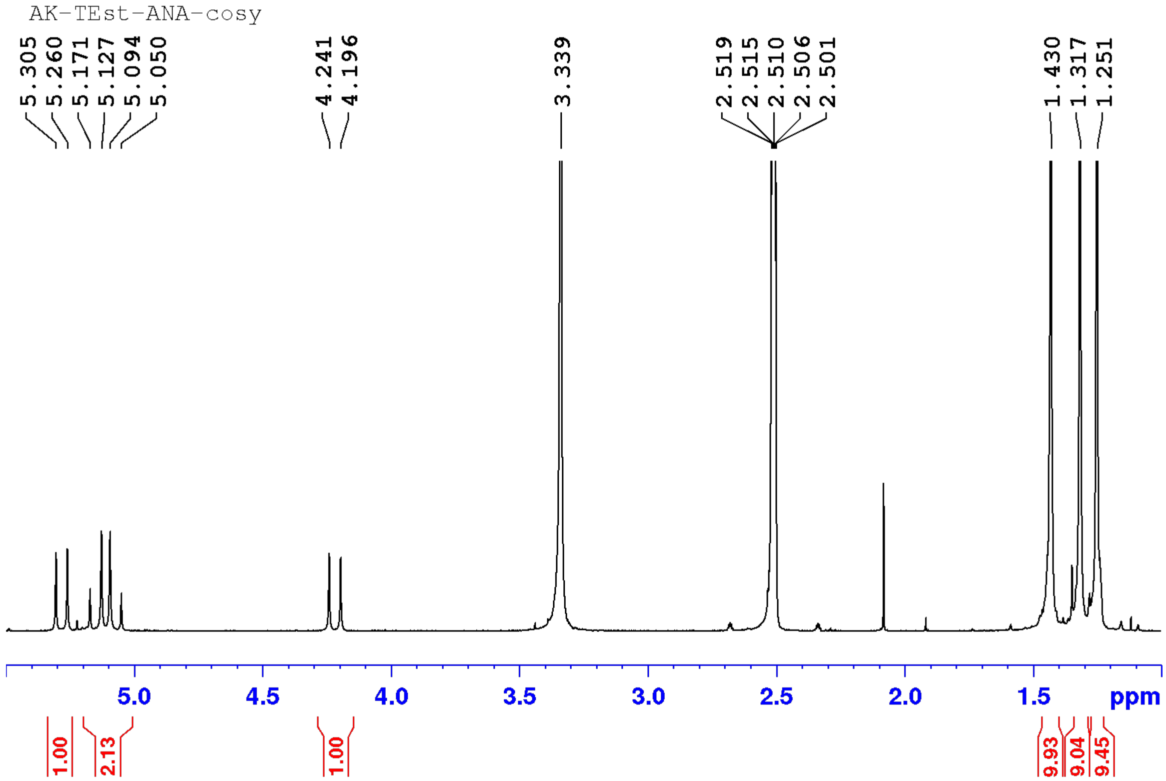

^{1}H-NMR spectrum of probe **1** in DMSO-d_6 (aliphatic region)

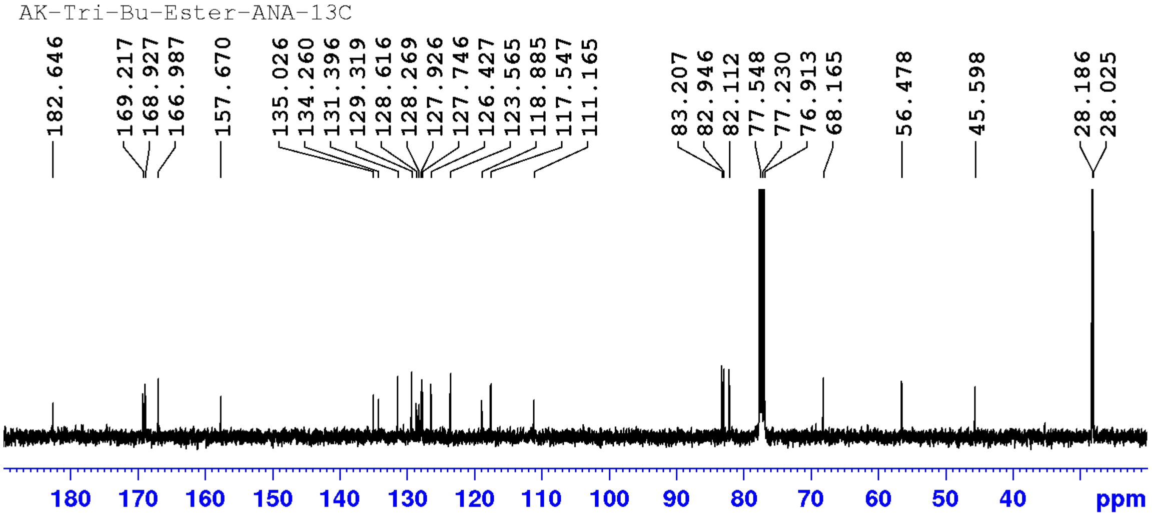

^{13}C-NMR spectrum of probe **1** in $CDCl_3$ (full)

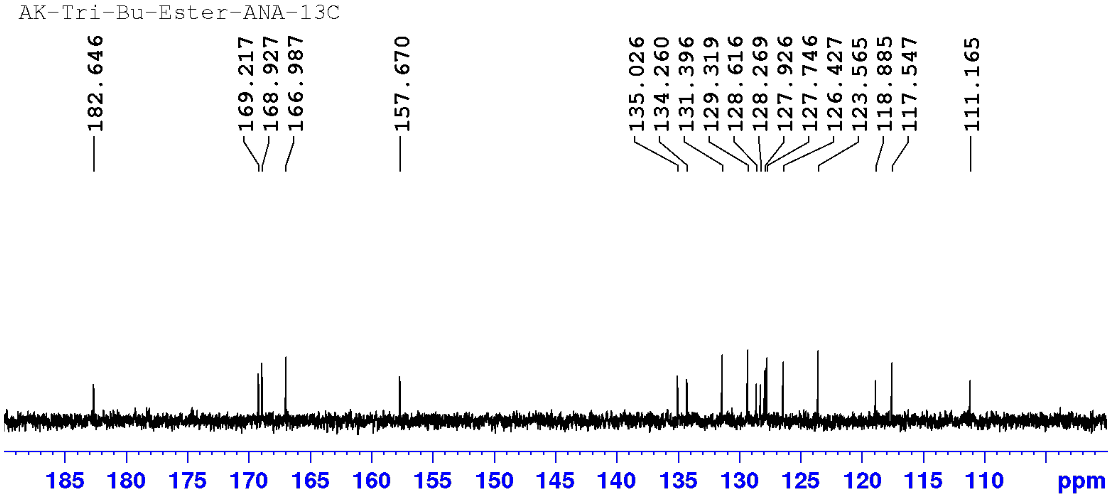

^{13}C-NMR spectrum of probe **1** in $CDCl_3$ (aromatic and carbonyl regions)

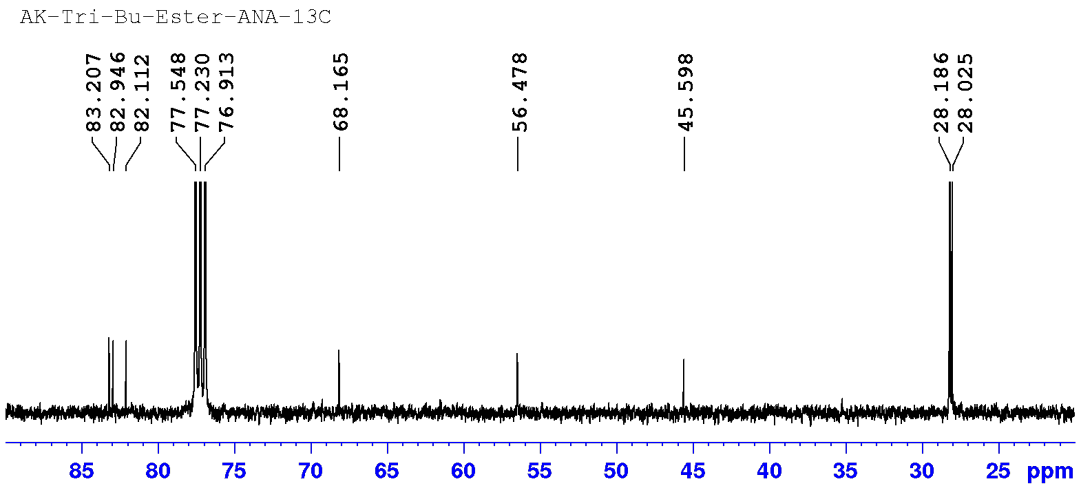

^{13}C-NMR spectrum of probe **1** in $CDCl_3$ (aliphatic region)

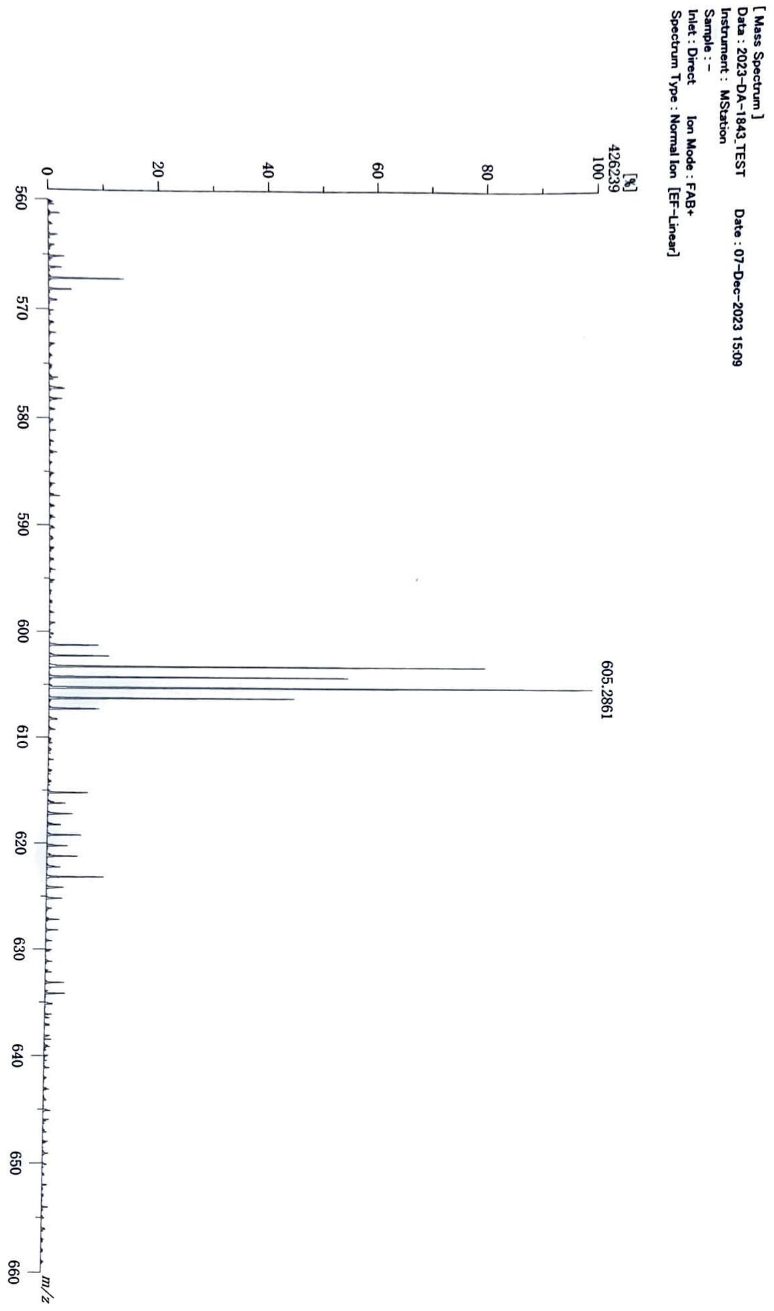

HRMS of probe **1**

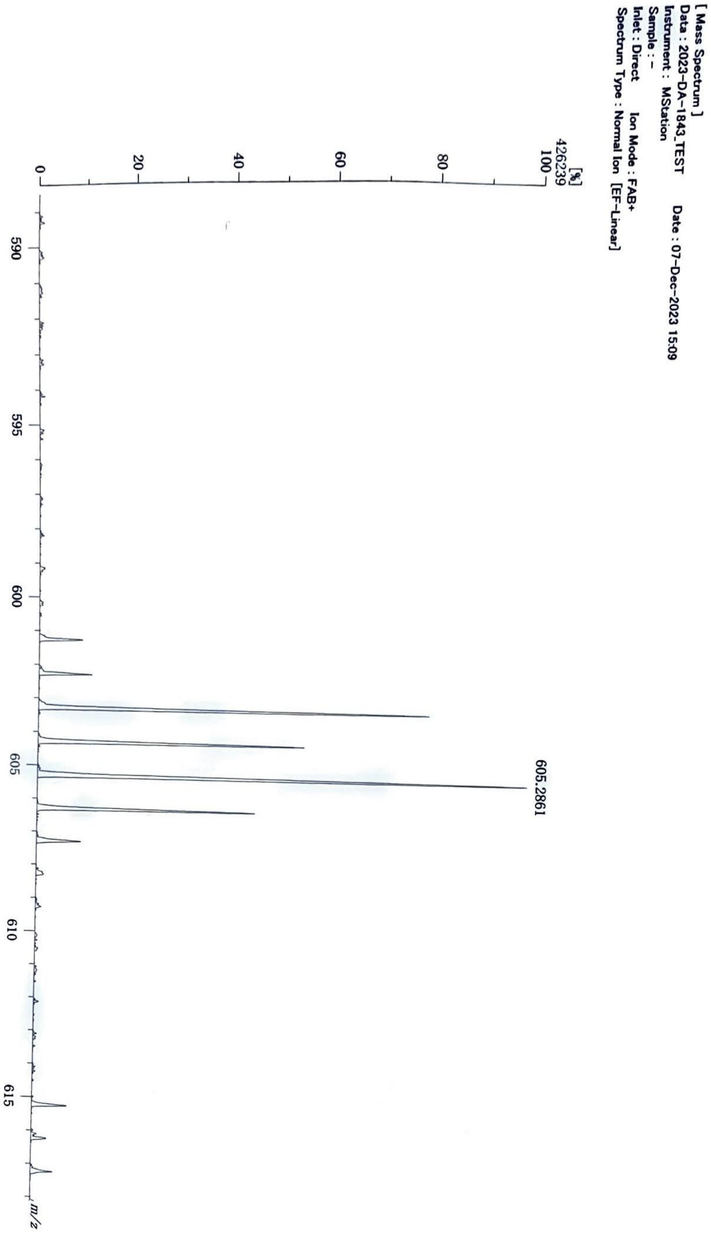

HRMS of probe **1**
